# Connection between *GW* and Extended Coupled Cluster

**DOI:** 10.1021/acs.jctc.6c00378

**Published:** 2026-06-29

**Authors:** Johannes Tölle, Marios-Petros Kitsaras, Andreas Irmler, Andreas Grüneis, Pierre-François Loos

**Affiliations:** † Department of Chemistry, 14915 University of Hamburg, Hamburg 22761, Germany; ‡ The Hamburg Centre for Ultrafast Imaging (CUI), Hamburg 22761, Germany; § Laboratoire de Chimie et Physique Quantiques (UMR 5626), 173637 Université de Toulouse, CNRS, Toulouse 31055, France; ∥ Institute for Theoretical Physics, 27259 TU Wien, Wiedner Hauptstraße 8-10/136, Vienna 1040, Austria

## Abstract

Coupled-cluster (CC) theory and Green’s function many-body perturbation theory (MBPT) have long evolved as distinct yet complementary frameworks for describing electronic correlation. While CC methods employ exponential wave function parametrizations that guarantee size extensivity and systematic improvability, Green’s function approaches, such as the *GW* approximation, describe quasiparticle and optical excitations through diagrammatic resummations. Recent analyses have established a formal correspondence between these frameworks: the *GW* approximation is equivalent to an equation-of-motion (EOM) treatment of the direct-ring coupled-cluster doubles (drCCD) method. Within this context, the extended CC (ECC) ansatz offers a unified framework connecting CC and MBPT. This formulation bridges CC-based and Green’s function-based methods, providing novel avenues for incorporating vertex corrections within a CC framework that preserves a sum-over-state representation of the self-energy and leads to potentially systematically improvable Green’s function approaches.

## Graphical abstract

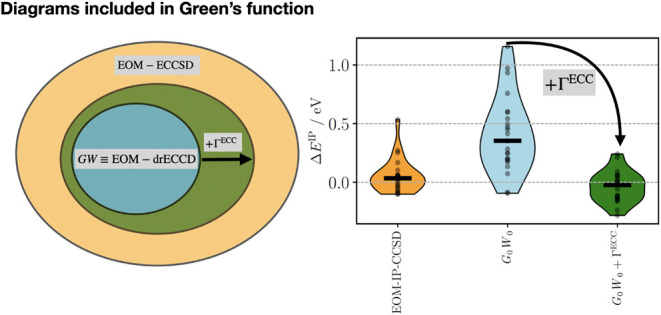

## 1 Introduction

Coupled-cluster (CC) theory is one of the most mature and powerful methodologies in electronic structure theory.^1–5^ Developed over several decades, it now provides access not only to highly accurate ground-state energetics and properties but also to excited states, through either the equation-of-motion (EOM) or linear-response formalisms.^6–10^ Thanks to the sustained efforts of many research groups, CC has become the reference method for high-accuracy electronic structure calculations in molecular systems.^11–21^

Inspired by the seminal work of Hubbard,^22^ the CC ansatz was originally introduced by Coester and Kümmel in the 1950s to describe correlations in nuclear matter.^23,24^ It was then brought into quantum chemistry in the 1960s through the seminal work of Sinanoğlu, Čížek, Paldus, and Shavitt.^25–27^ Interestingly, the method later migrated back to nuclear physics in the 1990s^28^ and is now widely used for accurate computations of atomic nuclei.^29–31^ More recently, CC has also found applications in condensed matter physics, where methodological and algorithmic advances have enabled its deployment in periodic solids.^32–40^

Decades of development have revealed both the strengths and limitations of CC theory. On the one hand, CC excels at describing ground- and excited-state properties of weakly correlated systems. The CCSD­(T) model, in particular, is widely regarded as the “gold standard” of quantum chemistry,^41,42^ with systematic extensions such as CCSDT(Q)^43^ providing a “platinum standard” for even higher accuracy.^44^ The introduction of the Lagrangian formalism by Helgaker and coworkers placed the computation of properties as analytic gradients on firm theoretical grounds, opening the way to accurate calculations of both static and frequency-dependent molecular properties of arbitrary order.^45–51^

On the other hand, the single-reference nature of traditional CC theory becomes problematic in multireference situations where the Hartree–Fock (HF) determinant is not a suitable starting point. In such cases, higher-rank excitations (triples, quadruples, etc.) are often required, an EOM-CC approach may be employed,^52–56^ or one must turn to one of the various flavors of multireference CC.^57–62^

A parallel story can be told for Green’s function many-body perturbation theory (MBPT).^63,64^ Inspired by nuclear physics, with early contributions from Salpeter and Bethe,^65^ Green’s function approaches became central in condensed matter physics in the 1960s. Hedin’s introduction of the *GW* approximation^66–68^ was a decisive turning point, as the concept of electronic screening proved remarkably successful in the description of the uniform electron gas.^69–71^ However, *GW* was not applied to realistic materials until the 1980s.^72–77^ While Green’s function techniques such as the algebraic diagrammatic construction (ADC)^78,79^ were already being developed for molecules in the 1970s by Cederbaum, Schirmer, von Niessen, Domcke, and coworkers,^80–87^ the transfer of the *GW* and Bethe–Salpeter equation (BSE) formalism to quantum chemistry occurred only more recently.^88–96^ This was partly because screening effects were thought to be less dominant in small- and medium-sized molecular systems. Today, however, both *GW* ^97,98^ and BSE^99,100^ are widely used in molecular science, complementing traditional wave function approaches.

In recent years, several groups have begun to explore the theoretical connections between CC theory and Green’s function MBPT. For example, Lange and Berkelbach analyzed the diagrammatic content of *GW* and ionization potential (IP) and electron affinity (EA) EOM-CC theory,^8,52,53,101–103^ identifying both striking similarities and key differences between the two.^104^ They showed that EOM-CC with singles and doubles (EOM-CCSD)^7,8,41,47,105,106^ contains fewer ring diagrams than *GW*, but incorporates a large number of vertex corrections (i.e., beyond ring diagrams) arising from ladder, mixed ring-ladder, and exchange diagrams. Including triples yields EOM-CCSDT,^101,107–111^ which includes all diagrams contained in the *GW* approximation, along with many additional high-order vertex corrections. Their work builds on earlier insights by Scuseria and coworkers,^112,113^ who related diagrammatic truncations of CC with doubles (CCD) to variants of the random-phase approximation (RPA) (see also refs 114–118 ).

More recently, Quintero-Monsebaiz et al. established connections between BSE@*GW* and CC at both the ground- and excited-state levels, enabling transfer of methodological insights from one framework to the other.^119^ They further showed that both *GW* and BSE can be recast as nonlinear CC-like equations solvable with the standard CC machinery with the same computational scaling. In the same spirit, Coveney and Tew have investigated the interconnections between various MBPT schemes and CCD, with a particular focus on the CC self-energy and Green’s function.^120–122^ Building on the “upfolded” version of *GW* introduced by Bintrim and Berkelbach,^123^ Tölle and Chan subsequently uncovered an exact connection between *GW* and the unitary CCD ansatz restricted to the direct-ring (dr) diagrams (drCCD).^124^ These developments have already borne fruit: One of the authors of the present study derived the first fully analytic *GW* nuclear gradients,^125^ and this work was recently extended by some of us to the first analytic BSE nuclear gradients.^126^ Nevertheless, this strategy requires the numerical evaluation of an infinite series of nested (anti)­commutators, which is not standard in conventional CC implementations. Unfortunately, direct application of EOM-CCD to *GW* remains hampered by missing correlation effects, as analyzed in refs 117,124 . Very recently, some of us introduced a reformulation of the *GW* formalism that builds upon the well-established EOM-drCCD framework,^127^ providing an alternative, fully analytic route to *GW* nuclear gradients. This modified EOM-CCD formulation restores the missing correlation effects inherent to traditional CCD-based approaches while maintaining a consistent and rigorous connection with the *GW* approximation.

In this context, the extended CC (ECC) ansatz of Arponen offers a promising path forward.^128^ ECC generalizes standard CC by introducing a bivariational framework,^128–131^ in which both excitation and de-excitation operators are optimized simultaneously (see also refs 132–134 ). This ansatz preserves size-extensivity while providing a (bi) variational energy functional, making it particularly well-suited for the evaluation of molecular properties. In particular, the bivariational structure ensures that the Hellmann–Feynman theorem can be directly applied, thereby simplifying the computation of response properties.^135–139^

Closely related to the ECC framework is the XCC approach,^140,141^ for which a straightforward EOM variant can be formulated.^132^ Both ECC and XCC employ a doubly similarity-transformed Hamiltonian, which introduces further low-lying correlation contributions, such as important third-order perturbation theory terms that are absent in conventional EOM-CCSD.^104,132^

Comparative benchmark studies of various CC ansätze, including ECC, have been performed by Cooper and Knowles,^142^ as well as Evangelista.^143^ Van Voorhis and Head-Gordon introduced the quadratic CCD method via a simplification of the ECCD equations to preserve the computational scaling of the traditional CCD.^144^ In the present contribution, we revisit the relationship between *GW* and ECC and demonstrate how the latter can provide a natural framework for embedding Green’s function formalisms within the CC machinery.

The present work is organized as follows. Sections 2 and 3 introduce the *GW* approximation using two complementary frameworks: Hedin’s approach and the electron-boson formulation, respectively. Section 4 briefly reviews the traditional CC energy functional and the extended CC approach, which employs a double similarity transformation that plays a central role in this work. Section 5 applies an ECC-like double similarity transformation to the electron-boson Hamiltonian for the *GW* approximation introduced in Section 3. This yields an ECC electron-boson Hamiltonian, which is represented in Fock space in Section 6, yielding an eigenvalue problem for charged excitation energies. Section 7 demonstrates the equivalence between the eigenvalue problem of the ECC electron-boson Hamiltonian (Section 6) and the *GW* supermatrix (Section 3). Section 8 identifies the terms in the ECC electron-boson formulation that correspond to vertex corrections beyond *GW*. In Section 9, we motivate a low-order approximation to the *GW* density matrix within the framework of the double similarity transformed Hamiltonian. The resulting linearized one-body density matrix yields an additional correction in the Fock matrix, which can be viewed as an approximation to self-consistent *GW*. Computational details are summarized in Section 10. Numerical results for principal and secondary IPs of a molecular test set are reported in Section 11, where different levels of theory and vertex corrections are compared. Finally, conclusions are drawn in Section 12.

## 2 The *GW* Approximation

The *GW* approximation is most rigorously formulated within the framework of Hedin’s equations,^66^ which constitute a self-consistent set of five coupled integro-differential relations connecting the one-body Green’s function *G*, the dynamically screened Coulomb interaction *W*, the irreducible vertex function Γ, the irreducible polarizability *P*, and the exchange-correlation (xc) self-energy Σ_xc_:

1a $$\Gamma \left(123\right)=\delta \left(12\right)\delta \left(13\right)+\frac{\delta \Sigma_{\mathrm{xc}}\left(12\right)}{\delta G\left(45\right)}G\left(46\right)G\left(75\right)\Gamma \left(673\right)$$

1b P(12)=−iG(13)G(41)Γ(342)

1c W(12)=v(12)+v(13)P(34)W(42)

1d $$\Sigma_{\mathrm{xc}}\left(12\right)=iG\left(14\right)W\left(1^{+}3\right)\Gamma \left(423\right)$$

1e $$G\left(12\right)=G_{H}\left(12\right)+G_{H}\left(13\right)\Sigma_{\mathrm{xc}}\left(34\right)G\left(42\right)$$

where *v* denotes the bare Coulomb interaction, and *G*_H_ is the Hartree Green’s function. Here, integer numbers denote combined space-spin-time variables.

The *GW* approximation arises upon replacing the three-point irreducible vertex Γ by its zeroth-order form, Γ(123) = δ(12)­δ(13), which neglects vertex corrections beyond the independent-particle response. Under this approximation, the irreducible polarizability reduces to

2 P(12)=−iG(12)G(21)

and the xc self-energy simplifies to

3 $$\Sigma_{\mathrm{xc}}\left(12\right)=iG\left(12\right)W\left(1^{+}2\right)$$

Systematic improvements beyond *GW* can then, in principle, be obtained by including vertex corrections in either *P* (internal corrections) and/or Σ_xc_ (external corrections) in combination with careful diagrammatic analysis to avoid incomplete diagrammatic expansions.^145–162^

It is worth noting that both *G* and *W* satisfy Dyson-like equations [see eq 3 and 4, respectively], with Σ_xc_ and *P* serving as their respective kernels. This observation highlights that the *GW* approximation is inherently a two-step procedure. In the first step, *P* is computed to determine the dynamical screening *W*. In the second step, Σ_xc_ is evaluated in order to obtain the one-body Green’s function *G*.

An alternative and particularly transparent way to view the *GW* approximation is through its supermatrix representation.^119,123,163–167^ In this formalism, *GW* can be written as a linear eigenvalue problem of the form

4 $$H^{GW}\cdot R=R\cdot E$$

where ***E*** gathers the charged excitation energies (quasiparticle and satellites), the *GW* supermatrix reads

5 $$H^{GW}=\left(\begin{array}{ccc} f & M^{2h1p} & M^{2p1h}\\ {\left(M^{2h1p}\right)}^{†} & C^{2h1p} & 0\\ {\left(M^{2p1h}\right)}^{†} & 0 & C^{2p1h} \end{array}\right)$$

and

6 $$R=\left(\begin{array}{c} r\\ r^{2h1p}\\ r^{2p1h} \end{array}\right)$$

Here, ***f*** is the Fock matrix containing the one-hole (1h) and one-particle (1p) configurations, and diagonal sub-blocks defined as

7 $$C_{i\nu ,i\nu }^{2h1p}=\epsilon_{i}-\Omega_{\nu },C_{a\nu ,a\nu }^{2p1h}=\epsilon_{a}+\Omega_{\nu }$$

for the two-hole-one-particle (2h1p) and two-particle-one-hole (2p1h) configurations, where ϵ_*p*_ are the quasiparticle energies. The corresponding coupling matrices,

8 $$M_{p,i\nu }^{2h1p}=M_{pi,\nu },M_{p,a\nu }^{2p1h}=M_{pa,\nu }$$

contain the effective two-electron integrals,

9 $$M_{pq,\nu }=\underset{ia}{\sum }\left\langle pa\right|qi\rangle (X+Y{)}_{ia,\nu }$$

where ***X*** and ***Y*** are the RPA excitation and deexcitation vectors associated with the one-hole-one-particle (1h1p) RPA (positive) excitation energies Ω_ν_ (see below). The two-electron integrals ⟨*pq*|*rs*⟩ are given in Dirac notation, i.e., ⟨12|12⟩. In this work, the indices *p*, *q*, *r*, *s*, ... are used for arbitrary orbitals, *i*, *j*, *k*, *l* label the occupied orbitals, *a*, *b*, *c*, *d* denote the virtual orbitals, and μ, ν, ... (collective) combined particle-hole indices. Furthermore, real-valued orbitals are assumed throughout.

The above linear eigenvalue problem can equivalently be recast as a nonlinear quasiparticle equation,

10 $$[f+\Sigma_{c}\left(\omega \right)-\omega 1]\cdot r=0$$

where the correlation self-energy takes the form

11 $$\begin{array}{cc} \Sigma_{c}\left(\omega \right) & =M^{2h1p}\cdot {\left(\omega 1-C^{2h1p}\right)}^{-1}\cdot {\left(M^{2h1p}\right)}^{†}\\ & +M^{2p1h}\cdot {\left(\omega 1-C^{2p1h}\right)}^{-1}\cdot {\left(M^{2p1h}\right)}^{†} \end{array}$$

The elements of the correlation part of the self-energy read

12 $$(\Sigma_{c}{)}_{pq}\left(\omega \right)=\underset{i\nu }{\sum }\frac{M_{pi,\nu }M_{qi,\nu }}{\omega -\epsilon_{i}+\Omega_{\nu }}+\underset{a\nu }{\sum }\frac{M_{pa,\nu }M_{qa,\nu }}{\omega -\epsilon_{a}-\Omega_{\nu }}$$

The matrices ***X*** and ***Y*** are obtained by solving the direct RPA eigenvalue problem,

13 $$\left(\begin{array}{cc} A & B\\ -B & -A \end{array}\right)\cdot \left(\begin{array}{cc} X & Y\\ Y & X \end{array}\right)=\left(\begin{array}{cc} X & Y\\ Y & X \end{array}\right)\cdot \left(\begin{array}{cc} \Omega & 0\\ 0 & -\Omega \end{array}\right)$$

where

14a $$A_{ia,jb}=\left(\epsilon_{a}-\epsilon_{i}\right)\delta_{ij}\delta_{ab}+\left\langle ib\right|aj\rangle$$

14b $$B_{ia,jb}=\left\langle ij\right|ab\rangle$$

Solving this RPA problem, which scales as $O{(N}^{6})$ , is a prerequisite for constructing the *GW* supermatrix. It can be shown to be equivalent to solving the Riccati equation^112^

15 B+A·t+t·A+t·B·t=0

with

16 $$t=Y\cdot X^{-1}$$

Although this formal equivalence is useful, it does not by itself provide a clear physical interpretation of the *GW* approximation. Nevertheless, it clearly shows that introducing vertex corrections within the same excitation manifold (i.e., up to 2h1p and 2p1h configurations) can only be achieved by modifying one or more of the following quantities in eq 9: the Fock matrix ***f***, the diagonal blocks ***C***, or the coupling blocks ***M***.

## 3 *GW* as an Electron-Boson Problem

A complementary and highly insightful perspective on the *GW* approximation is obtained by recasting it as an electron-boson coupling model.^165,168–171^ Note that this effectively corresponds to a “coarse-graining” of electronic degrees of freedom into quasi-bosonic excitations, a common approximation in the context of boson expansion techniques.^172^ Within this picture, an electron added to or removed from the system interacts linearly with a bath of bosonic excitations representing the collective electronic response.^165^ In this framework, the RPA excitations are interpreted as effective bosonic modes that mediate the dynamical screening of the Coulomb interaction. The resulting Hamiltonian reads

17 $${Ĥ}_{\mathrm{eB}}={Ĥ}_{e}+{Ĥ}_{B}+{\mathrm{V̂}}_{\mathrm{eB}}$$

where

18a $${Ĥ}_{e}=\underset{pq}{\sum }f_{pq}{â}_{p}^{†}{â}_{q}$$

18b $${Ĥ}_{B}=\underset{\mu \nu }{\sum }A_{\mu \nu }{\mathrm{b̂}}_{\mu }^{†}{\mathrm{b̂}}_{\nu }+\frac{1}{2}\underset{\mu \nu }{\sum }B_{\mu \nu }\left({\mathrm{b̂}}_{\mu }^{†}{\mathrm{b̂}}_{\nu }^{†}+{\mathrm{b̂}}_{\mu }{\mathrm{b̂}}_{\nu }\right)$$

18c $${\mathrm{V̂}}_{\mathrm{eB}}=\underset{pq\nu }{\sum }V_{pq,\nu }{â}_{p}^{†}{â}_{q}\left({\mathrm{b̂}}_{\nu }^{†}+{\mathrm{b̂}}_{\nu }\right)$$

respectively describe the Fermionic subsystem, the bosonic bath, and their mutual coupling. Here, *f*_*pq*_ is an element of the Fock matrix, *V*_*pq*,ν_ ≡ *V*_*pq*,*ia*_ = ⟨*pa*|*qi*⟩ are two-electron integrals that mediate the coupling between the electronic and bosonic degrees of freedom, and the elements of the particle-conserving and particle-nonconserving components of the bosonic Hamiltonian are given by [see eq 18–19]­

19 $$A_{\mu \nu }\equiv A_{ia,jb},B_{\mu \nu }\equiv B_{ia,jb}$$

Although neutral electronic excitations are fundamentally composed of Fermions, they can often be treated approximately as bosons. In this framework, quasiboson creation and annihilation operators emulate Fermionic particle-hole excitations,

20 $${\mathrm{b̂}}_{\nu }^{†}={â}_{a}^{†}{â}_{i},{\mathrm{b̂}}_{\nu }={â}_{i}^{†}{â}_{a}$$

where ${â}_{a}^{†}$ and *â*_*i*_ denote Fermionic creation and annihilation operators, respectively. Because of their underlying Fermionic structure, these quasiboson operators do not strictly satisfy Fermionic anticommutation relations anymore, and therefore violate the Pauli exclusion principle and the antisymmetry of the exact electronic wave function.^112^ The quasiboson approximation consists in neglecting these deviations and treating quasibosons as ideal bosons. This approximation not only simplifies the algebra and the physical interpretation of excitation processes but also transforms otherwise *quartic* Fermionic operators, such as the two-body part of the electronic Hamiltonian, into effective (bosonized) *quadratic* Hamiltonians in the quasiboson operators.

Using the composite basis made of the union of the 1h {*â*_*i*_}, 1p {*â*_*a*_}, $2\mathrm{h1p}\left\{{â}_{i}{\mathrm{b̂}}_{\nu }^{†}\right\}$ , and 2p1h {*â*_*a*_ *b̂*_ν_} configurations, an IP/EA-EOM treatment on the electron-boson Hamiltonian naturally yields the *GW* supermatrix within the Tamm-Dancoff approximation (TDA).^123^

To go beyond the TDA, the bosonic Hamiltonian must be diagonalized through a Bogoliubov transformation, which is conveniently expressed as^165^

$${Ĥ}_{B}=-\frac{1}{2}\mathrm{Tr}\;A+\frac{1}{2}\left(\begin{array}{cc} b^{†} & b \end{array}\right)\cdot \left(\begin{array}{cc} A & B\\ B & A \end{array}\right)\cdot \left(\begin{array}{c} b\\ b^{†} \end{array}\right)$$ 21

Introducing the quasiparticle (Bogoliubov) operators^172^

22 $$\left(\begin{array}{c} \beta \\ \beta^{†} \end{array}\right)={\left(\begin{array}{cc} X & -Y\\ -Y & X \end{array}\right)}^{†}\cdot \left(\begin{array}{c} b\\ b^{†} \end{array}\right)$$

defined through a unitary transformation built from the RPA eigenvectors, the Hamiltonian becomes

$${Ĥ}_{B}=-\frac{1}{2}\mathrm{Tr}\;A+\frac{1}{2}\left(\begin{array}{cc} \beta^{†} & \beta \end{array}\right)\cdot \left(\begin{array}{cc} \Omega & 0\\ 0 & \Omega \end{array}\right)\cdot \left(\begin{array}{c} \beta \\ \beta^{†} \end{array}\right)$$ 23

The components of the Hamiltonian can then be recast as

24a $${Ĥ}_{B}=E_{c}+\underset{\mu }{\sum }\Omega_{\mu }{\mathrm{\beta ̂}}_{\mu }^{†}{\mathrm{\beta ̂}}_{\mu }$$

24b $${\mathrm{V̂}}_{\mathrm{eB}}=\underset{\mu \nu }{\sum }M_{pq,\nu }{â}_{p}^{†}{â}_{q}\left({\mathrm{\beta ̂}}_{\nu }^{†}+{\mathrm{\beta ̂}}_{\nu }\right)$$

where the RPA correlation energy is

25 $$E_{c}=\frac{1}{2}\mathrm{Tr}\left(\Omega -A\right)$$

The particle-nonconserving bosonic terms in *Ĥ*_B_ are thus removed by the Bogoliubov transformation that diagonalizes the RPA problem. However, full diagonalization is not strictly necessary: a block-diagonalization already suffices to decouple excitation and deexcitation subspaces, rendering the unitary Bogoliubov transformation somewhat excessive. In the following sections, we will first introduce the extended CC approach and then show that an equivalent block-diagonal structure can be achieved more directly through a double similarity transformation.

## 4 Extended Coupled Cluster

The traditional CC (TCC) energy functional is

26 $$E_{\mathrm{TCC}}=\left\langle \Phi_{0}\right|\left(1+\mathrm{\Lambda ̂}\right)e^{-\mathrm{T̂}}Ĥe^{\mathrm{T̂}}\left|\Phi_{0}\right\rangle$$

where *T̂* = ∑_*q*_ *t*_*q*_ *τ̂*_*q*_ is an excitation operator written as a function of the general excitation operator *τ̂*_*q*_ and $\mathrm{\Lambda ̂}=\underset{q}{\sum }\lambda_{q}{\mathrm{\tau ̂}}_{q}^{†}$ is a deexcitation operator. These operators act on the reference wave function Φ_0_ to generate excited determinant Φ_*q*_ as |Φ_*q*_⟩ = *τ̂*_*q*_|Φ_0_⟩ and $\left\langle \Phi_{q}\right|=\left\langle \Phi_{0}\right|{\mathrm{\tau ̂}}_{q}^{†}$ .

The extended CC energy bifunctional is

27 $$E_{\mathrm{ECC}}=\left\langle \Phi_{0}\right|e^{Ẑ}e^{-\mathrm{T̂}}Ĥe^{\mathrm{T̂}}e^{-Ẑ}\left|\Phi_{0}\right\rangle$$

where the linear operator 1 + Λ̂ has been replaced by a proper exponential operator $e^{Ẑ}$ with $Ẑ=\underset{q}{\sum }z_{q}{\mathrm{\tau ̂}}_{q}^{†}$ . In other words, TCC can be seen as an approximation of ECC. Making the ECC energy functional stationary with respect to the right amplitudes *t*_*q*_ and left amplitudes *z*_*q*_, i.e.,

28 $$\frac{\partial E_{\mathrm{ECC}}}{\partial z_{q}}=0,\frac{\partial E_{\mathrm{ECC}}}{\partial t_{q}}=0$$

yields the amplitude equations

29a $$\left\langle \Phi_{q}\right|e^{Ẑ}e^{-\mathrm{T̂}}Ĥe^{\mathrm{T̂}}\left|\Phi_{0}\right\rangle =0$$

29b $$\left\langle \Phi_{0}\right|e^{Ẑ}e^{-\mathrm{T̂}}\left[Ĥ,{\mathrm{\tau ̂}}_{q}\right]e^{\mathrm{T̂}}\left|\Phi_{0}\right\rangle =0$$

which, contrary to TCC, couple the two sets of amplitudes. Note that an alternative formulation of the ECC amplitude equations through projection exists,^132^ and numerical results for ground-state energies using both schemes have been shown to be similar.^133,134^

When restricted to double excitations, that is, *T̂* = *T̂*_2_, the ECC energy functional becomes

30 $$\begin{array}{ll} E_{\mathrm{ECC}} & =\left\langle \Phi_{0}\right|e^{Ẑ}(Ĥe^{{\mathrm{T̂}}_{2}}{)}_{c}\left|\Phi_{0}\right\rangle \\ & =\left\langle \Phi_{0}\right|e^{Ẑ}(Ĥ+Ĥ{\mathrm{T̂}}_{2}+\frac{1}{2}Ĥ{\mathrm{T̂}}_{2}^{2}+\frac{1}{6}Ĥ{\mathrm{T̂}}_{2}^{3}+\frac{1}{24}Ĥ{\mathrm{T̂}}_{2}^{4}{)}_{c}\left|\Phi_{0}\right\rangle \\ & =\left\langle \Phi_{0}\right|Ĥ+Ẑ(Ĥ+Ĥ{\mathrm{T̂}}_{2}+\frac{1}{2}Ĥ{\mathrm{T̂}}_{2}^{2}{)}_{c}\\ & +\frac{1}{2}{Ẑ}^{2}(\frac{1}{2}Ĥ{\mathrm{T̂}}_{2}^{2}+\frac{1}{6}Ĥ{\mathrm{T̂}}_{2}^{3}{)}_{c}+\frac{1}{6}{Ẑ}^{3}(\frac{1}{24}Ĥ{\mathrm{T̂}}_{2}^{4}{)}_{c}\left|\Phi_{0}\right\rangle \end{array}$$

The subscript c stands for “connected” and replaces (nested) commutator(s) between the Hamiltonian and the cluster operator, e.g., [*Ĥ*, *T̂*_2_] = (*ĤT̂*_2_)_c_, as only connected terms survive in these contributions.

At this stage, it is useful to emphasize the similarities and differences between the unitary and extended CC ansätze. The ECC form, $e^{Ẑ}e^{-\mathrm{T̂}}Ĥe^{\mathrm{T̂}}e^{-Ẑ}$ , can be recast as $e^{-\mathrm{t̂}}Ĥe^{\mathrm{t̂}}$ by defining $\mathrm{t̂}=\mathrm{T̂}-Ẑ+\frac{1}{2}\left[Ẑ,\mathrm{T̂}\right]+\cdot \cdot \cdot$ , sharing some resemblance with the unitary CC (UCC) expression with *t̂* = *T̂* – *T̂* ^†^. In UCC, the anti-Hermiticity condition *t̂* ^†^ = −*t̂* ensures a unitary transformation. In contrast, ECC does not enforce this constraint: *Ẑ* is independent of *T̂*, making *t̂* generally non-Hermitian, offering greater flexibility at the cost of Hermiticity. Unlike the UCC functional, which results in a nonterminating Baker–Campbell–Hausdorff (BCH) expansion, the ECC approach results in a terminating series, although at a much higher order than the traditional CCD functional. Lastly, we mention the XCC parametrization that bears the same truncating doubly similarity transformed Hamiltonian form as ECC but restricts the deexcitations to be the complex conjugate of the excitations *Ẑ* = *T̂* ^†^.^132^ It is argued that due to the decreased flexibility of the XCC ansatz, ECC is expected to recover Hermiticity more effectively in comparison.^132^

The computational cost of traditional CCD is $O{(N}^{6})$ , whereas ECCD scales as $O{(N}^{10})$ , which largely explains why it has not been widely adopted despite its attractive formal properties. Henderson and Scuseria demonstrated that the pair-ECCD method achieves the same $O{(N}^{3})$ scaling as the corresponding pair-CCD approach.^173,174^ Likewise, van Voorhis and Head-Gordon showed that truncating $e^{Ẑ}$ after the quadratic term reduces the scaling to $O{(N}^{6})$ .^144^ These results suggest that suitable restrictions on the double excitation operator can significantly lower the computational cost of ECCD. Below, we will show that the quadratic nature of the bosonic Hamiltonian underlying *GW* results in a significant simplification of both the working equations and the associated computational cost.

## 5 ECC on the Electron-Boson Hamiltonian

In this section, we apply the ECC ansatz to the bosonic Hamiltonian *Ĥ*_B_ defined in eq 24. As outlined in Section 4, a double similarity transformation is performed:

31 $${\mathrm{H̿}}_{B}=e^{Ẑ}{\mathrm{H̅}}_{B}e^{-Ẑ}=e^{Ẑ}e^{-\mathrm{T̂}}{Ĥ}_{B}e^{\mathrm{T̂}}e^{-Ẑ}$$

where the bosonic excitation and de-excitation operators are defined as

32 $$\mathrm{T̂}=\frac{1}{2}\underset{\mu \nu }{\sum }t_{\mu \nu }{\mathrm{b̂}}_{\mu }^{†}{\mathrm{b̂}}_{\nu }^{†},Ẑ=\frac{1}{2}\underset{\mu \nu }{\sum }z_{\mu \nu }{\mathrm{b̂}}_{\mu }{\mathrm{b̂}}_{\nu }$$

Because *Ĥ*_B_ is quadratic, the BCH expansion associated with the first similarity transformation truncates exactly at second order, yielding

33 $${\mathrm{H̅}}_{B}={Ĥ}_{B}+\left[{Ĥ}_{B},\mathrm{T̂}\right]+\frac{1}{2}\left[[{Ĥ}_{B},\mathrm{T̂}\right],\mathrm{T̂}]=\frac{1}{2}\underset{\lambda \sigma }{\sum }B_{\lambda \sigma }t_{\lambda \sigma }+\underset{\mu \nu }{\sum }{\mathrm{A̅}}_{\mu \nu }{\mathrm{b̂}}_{\mu }^{†}{\mathrm{b̂}}_{\nu }+\frac{1}{2}\underset{\mu \nu }{\sum }{\mathrm{B̅}}_{\mu \nu }{\mathrm{b̂}}_{\mu }^{†}{\mathrm{b̂}}_{\nu }^{†}\;+\frac{1}{2}\underset{\mu \nu }{\sum }B_{\mu \nu }{\mathrm{b̂}}_{\mu }{\mathrm{b̂}}_{\nu }$$

with

34a $${\mathrm{A̅}}_{\mu \nu }=A_{\mu \nu }+\underset{\lambda }{\sum }t_{\nu \lambda }B_{\lambda \mu }$$

34b $${\mathrm{B̅}}_{\mu \nu }=B_{\mu \nu }+\underset{\lambda }{\sum }A_{\mu \lambda }t_{\lambda \nu }+\underset{\lambda }{\sum }t_{\mu \lambda }A_{\lambda \nu }+\underset{\lambda \sigma }{\sum }t_{\mu \lambda }B_{\lambda \sigma }t_{\sigma \nu }$$

where we made use of the fact that ***t*** ^*T*^ = ***t***.^112^ As seen in eq 42, *H̅*_B_ remains quadratic, containing both number-conserving and non-number-conserving terms. Hence, the second BCH expansion also terminates at second order, leading to

35 $$\begin{array}{ll} {\mathrm{H̿}}_{B} & ={\mathrm{H̅}}_{B}+\left[Ẑ,{\mathrm{H̅}}_{B}\right]+\frac{1}{2}\left[Ẑ,\left[Ẑ,{\mathrm{H̅}}_{B}\right]\right]\\ & =\frac{1}{2}\underset{\lambda \sigma }{\sum }{\mathrm{B̅}}_{\lambda \sigma }z_{\lambda \sigma }+\underset{\mu \nu }{\sum }{\mathrm{A̿}}_{\mu \nu }{\mathrm{b̂}}_{\mu }^{†}{\mathrm{b̂}}_{\nu }\\ & +\frac{1}{2}\underset{\mu \nu }{\sum }{\mathrm{B̅}}_{\mu \nu }{\mathrm{b̂}}_{\mu }^{†}{\mathrm{b̂}}_{\nu }^{†}+\frac{1}{2}\underset{\mu \nu }{\sum }{\mathrm{B̿}}_{\mu \nu }{\mathrm{b̂}}_{\mu }{\mathrm{b̂}}_{\nu } \end{array}$$

where

36a $${\mathrm{A̿}}_{\mu \nu }={\mathrm{A̅}}_{\mu \nu }+\underset{\lambda }{\sum }z_{\nu \lambda }{\mathrm{B̅}}_{\lambda \mu }$$

36b $${\mathrm{B̿}}_{\mu \nu }=B_{\mu \nu }+\underset{\lambda }{\sum }{\mathrm{A̅}}_{\mu \lambda }z_{\lambda \nu }+\underset{\lambda }{\sum }z_{\mu \lambda }{\mathrm{A̅}}_{\lambda \nu }+\underset{\lambda \sigma }{\sum }z_{\mu \lambda }{\mathrm{B̅}}_{\lambda \sigma }z_{\sigma \nu }$$

Let |0_B_⟩ denote the bosonic reference vacuum. The right amplitude equations, obtained as ⟨0_B_| *b̂*_μ_ *b̂*_ν_ *H̿*_B_ |0_B_⟩ = 0, yield the condition *B̅*_*μν*_ = 0, which is equivalent to the (quadratic) Riccati equation in eq 20. The left amplitude equations, $\left\langle 0_{B}\right|{\mathrm{H̿}}_{B}{\mathrm{b̂}}_{\mu }^{†}{\mathrm{b̂}}_{\nu }^{†}\left|0_{B}\right\rangle =0$ , give *B̿*_*μν*_ = 0, which reduces to a set of linear equations in *z*_*μν*_ once *B̅*_*μν*_ = 0:

37 $$B_{\mu \nu }+\underset{\lambda }{\sum }{\mathrm{A̅}}_{\mu \lambda }z_{\lambda \nu }+\underset{\lambda }{\sum }z_{\mu \lambda }{\mathrm{A̅}}_{\lambda \nu }=0$$

Note that, although left- and right-amplitude equations are generally coupled, they are completely decoupled in the present case.

Once these two conditions are satisfied, the double similarity-transformed bosonic Hamiltonian simplifies to

38 $${\mathrm{H̿}}_{B}=\underset{\mu \nu }{\sum }{\mathrm{A̅}}_{\mu \nu }{\mathrm{b̂}}_{\mu }^{†}{\mathrm{b̂}}_{\nu }+\frac{1}{2}\underset{\lambda \sigma }{\sum }B_{\lambda \sigma }t_{\lambda \sigma }$$

where all non-number-conserving terms have been eliminated. This results in the block-diagonalization of the bosonic Hamiltonian, as discussed in Section 3.^127^

Having established the ECC treatment of the bosonic Hamiltonian, we can now turn to the full electron-boson Hamiltonian *Ĥ*_eB_ given in eq 22. The double similarity-transformed electron-boson Hamiltonian reads

39 $$\begin{array}{ll} {\mathrm{H̿}}_{\mathrm{eB}} & =e^{Ẑ}e^{-\mathrm{T̂}}{Ĥ}_{\mathrm{eB}}e^{\mathrm{T̂}}e^{-Ẑ}\\ & ={\mathrm{H̅}}_{\mathrm{eB}}+\left[Ẑ,{\mathrm{H̅}}_{\mathrm{eB}}\right]\\ & ={Ĥ}_{e}+{\mathrm{H̿}}_{B}+{\mathrm{V̿}}_{\mathrm{eB}} \end{array}$$

with

40 $$\begin{array}{ll} {\mathrm{V̿}}_{\mathrm{eB}} & =\underset{pq}{\sum }\underset{\nu }{\sum }V_{pq,\nu }{â}_{p}^{†}{â}_{q}\left({\mathrm{b̂}}_{\nu }^{†}+{\mathrm{b̂}}_{\nu }\right)\\ & +\underset{pq}{\sum }\underset{\nu }{\sum }\underset{\lambda }{\sum }V_{pq,\lambda }t_{\lambda \nu }{â}_{p}^{†}{â}_{q}{\mathrm{b̂}}_{\nu }^{†}\\ & +\underset{pq}{\sum }\underset{\nu }{\sum }\left[\underset{\lambda }{\sum }V_{pq,\lambda }z_{\lambda \nu }+\underset{\mu \lambda }{\sum }V_{pq,\lambda }t_{\lambda \mu }z_{\mu \nu }\right]{â}_{p}^{†}{â}_{q}{\mathrm{b̂}}_{\nu } \end{array}$$

## 6 EOM on the ECC Electron-Boson Hamiltonian

Building on the ECC electron-boson Hamiltonian introduced in Section 5, we next derive the EOM formulation for charged excitations. Within the present ECC-based formalism, the excitation operator *R̿* ^(*m*)^ for the *m*th excited state is defined as

41 $$\begin{array}{ll} {\mathrm{R̿}}^{\left(m\right)} & =e^{\mathrm{T̂}}e^{-Ẑ}{\mathrm{R̂}}^{\left(m\right)}e^{Ẑ}e^{-\mathrm{T̂}}\\ & =\underset{M}{\sum }R_{M}^{\left(m\right)}{c̿}_{M}=\underset{M}{\sum }R_{M}^{\left(m\right)}e^{\mathrm{T̂}}e^{-Ẑ}{ĉ}_{M}e^{Ẑ}e^{-\mathrm{T̂}}\\ & =\underset{i}{\sum }r_{i}^{\left(m\right)}a_{i}+\underset{i\nu }{\sum }r_{i\nu }^{\left(m\right)}{\mathrm{b̿}}_{\nu }^{†}a_{i}+\underset{a}{\sum }r_{a}^{\left(m\right)}a_{a}+\underset{a\nu }{\sum }r_{\nu a}^{\left(m\right)}{\mathrm{b̿}}_{\nu }a_{a} \end{array}$$

where *ĉ*_*M*_ denotes an excitation operator associated with the *M*th excitation process [see eq 10]. The various excitation channels are expressed in terms of double similarity-transformed bosonic creation and annihilation operators,

42a $${\mathrm{b̿}}_{\nu }^{†}=e^{\mathrm{T̂}}e^{-Ẑ}{\mathrm{b̂}}_{\nu }^{†}e^{Ẑ}e^{-\mathrm{T̂}}$$

42b $${\mathrm{b̿}}_{\nu }=e^{\mathrm{T̂}}e^{-Ẑ}{\mathrm{b̂}}_{\nu }e^{Ẑ}e^{-\mathrm{T̂}}$$

as well as the corresponding left and right reference states in the combined Fermionic-bosonic space,

43a $$\left|0_{e}{0̿}_{B}\right\rangle =e^{\mathrm{T̂}}e^{-Ẑ}\left|0_{e}0_{B}\right\rangle$$

43b $$\left\langle 0_{e}{0̿}_{B}\right|=\left\langle 0_{e}0_{B}\right|e^{Ẑ}e^{-\mathrm{T̂}}$$

The use of these transformed creation and annihilation operators is essential to ensure that the EOM formalism satisfies the required “killer” conditions.^175–180^

The EOM eigenvalue problem becomes

44 $$\left\langle 0_{e}{0̿}_{B}\right|\left[{c̿}_{M}^{†},\left[{Ĥ}_{\mathrm{eB}},{\mathrm{R̿}}^{\left(m\right)}\right]\right]\left|0_{e}{0̿}_{B}\right\rangle =\left\langle 0_{e}{0̿}_{B}\right|\left[{c̿}_{M}^{†},{\mathrm{R̿}}^{\left(m\right)}\right]\left|0_{e}{0̿}_{B}\right\rangle E^{\left(m\right)}$$

with *E* ^(*m*)^ denoting the energy of the *m*th charged excited state, and ${c̿}_{M}^{†}$ to the *M*th excitation/deexcitation process [see eq 52], respectively. eq 57 can be simplified to

45 $$\left\langle 0_{e}0_{B}\right|\left[{ĉ}_{M}^{†},\left[{\mathrm{H̿}}_{\mathrm{eB}},{\mathrm{R̂}}^{\left(m\right)}\right]\right]\left|0_{e}0_{B}\right\rangle =\left\langle 0_{e}0_{B}\right|\left[{ĉ}_{M}^{†},{\mathrm{R̂}}^{\left(m\right)}\right]\left|0_{e}0_{B}\right\rangle E^{\left(m\right)}$$

and the resulting EOM eigenvalue problem can be written as

46 $$H^{\mathrm{EOM}}\cdot R=R\cdot E$$

The equivalence with the *G_0_W*_0_ quasiparticle energies is established analytically in Section 7, identifying the matrix elements of the effective Hamiltonian ***H*** ^EOM^ with those of the *GW* supermatrix ***H*** ^*GW*^ defined in eq 9.

Details regarding the determination of analytic properties within the ECC treatment of the electron-boson Hamiltonian can be found in ref 127.

## 7 Equivalence with *G*_0_ *W*_0_

Having derived the EOM quasiparticle equations using the ECC electron-boson Hamiltonian [see eq 59], we now establish their analytical equivalence with the *G*_0_ *W*_0_ quasiparticle equations. For this, we rewrite the effective Hamiltonian ***H*** ^EOM^ in block form as

47 $$H^{\mathrm{EOM}}=\left(\begin{array}{ccc} f & {Ñ}^{2h1p} & N^{2p1h}\\ {\left(N^{2h1p}\right)}^{†} & D^{2h1p} & 0\\ {\left({Ñ}^{2p1h}\right)}^{†} & 0 & D^{2p1h} \end{array}\right)$$

The matrices read

48a $${[{Ñ}^{2h1p}]}_{pi,\mu }=\underset{\nu }{\sum }V_{pi,\nu }\left(\delta_{\nu \mu }+z_{\nu \mu }+\underset{\lambda }{\sum }t_{\nu \lambda }z_{\lambda \mu }\right)$$

48b $${[{Ñ}^{2p1h}]}_{pa,\mu }=\underset{\nu }{\sum }V_{pa,\nu }\left(\delta_{\nu \mu }+z_{\nu \mu }+\underset{\lambda }{\sum }t_{\nu \lambda }z_{\lambda \mu }\right)$$

48c $${[N^{2h1p}]}_{pi,\mu }=\underset{\nu }{\sum }V_{pi,\nu }\left(\delta_{\nu \mu }+t_{\nu \mu }\right)$$

48d $${[N^{2p1h}]}_{pa,\mu }=\underset{\nu }{\sum }V_{pa,\nu }\left(\delta_{\nu \mu }+t_{\nu \mu }\right)$$

and

49a $${[D^{2h1p}]}_{i\nu ,j\mu }=f_{ij}-A_{\nu \mu }-\underset{\lambda }{\sum }t_{\nu \lambda }B_{\lambda \mu }$$

49b $${[D^{2p1h}]}_{a\nu ,b\mu }=f_{ab}+A_{\nu \mu }+\underset{\lambda }{\sum }B_{\nu \lambda }t_{\lambda \mu }$$

To demonstrate the equivalence of eq 59 with the *G*_0_ *W*_0_ supermatrix [see eq 9], we begin by establishing the following relation (**1** + ***t***)· ***X*** = ***X*** + ***Y***, which follows directly from eq 21. Furthermore, we note that **1** + ***z*** + ***t*** · ***z*** = (**1** – ***t***)^−1^ (see the Supporting Information), simplifying eqs 61 and 62. From these relations, one finds that (**1** – ***t***)^−1^· ***X*** ^–1^ = (***X*** – ***Y***)^−1^ = ***X*** + ***Y***, where we made use of ***t*** ^*T*^ = ***t***.^112^ Finally, one can show that^127^

50 $$X\cdot \left(A+t\cdot B\right)\cdot X^{-1}=\Omega$$

Using the relations derived above, the EOM-ECC eigenvalue problem can be rewritten as

51 $$H^{\mathrm{EOM}}\cdot S^{-1}\cdot S\cdot R=R\cdot E$$

which, after multiplication from the left by the metric

52 $$S=\left(\begin{array}{ccc} 1 & 0 & 0\\ 0 & 1^{h}⊗X & 0\\ 0 & 0 & 1^{p}⊗X^{-1} \end{array}\right)$$

is identical to the *G*_0_ *W*_0_ supermatrix formulation of refs 119,123,163–167 and results in the effective Hamiltonian ***H*** ^*GW*^ of eq 9.

Elements of the transformation matrix ***S*** are given by

53 $${[1^{h}⊗X]}_{k\nu ,j(ia)}=\delta_{kj}X_{ia,\nu }$$

in the hole-quasiboson block, and

54 $${[1^{p}⊗X^{-1}]}_{\nu c,(ia)b}=\delta_{bc}(X^{-1}{)}_{\nu ,ia}$$

in the particle-quasiboson block. We have explicitly written down the matrix elements with combined occupied and virtual indices (*ia*) to avoid confusion with collective particle-hole indices ν arising from transformation in the excitation basis through ***X***.

## 8 Beyond-*GW* Vertex Corrections from ECC

In our opinion, the newly established connection between the ECC formalism and the *GW* approximation opens exciting avenues for systematically including vertex corrections beyond *GW*. As noted at the end of Section 2, vertex corrections can enter through three distinct components of the *GW* supermatrix [see eq 9]: (i) the Fock matrix ***f***, (ii) the diagonal blocks ***C***, and (iii) the coupling blocks ***M***. Reformulating the building blocks ***C*** and ***M*** within the ECC framework [see eq 60] provides direct access to vertex corrections that preserve the sum-over-state representation of the underlying self-energy. While such a sum-over-state representation is directly linked to causal self-energies for Hermitian effective Hamiltonians,^181^ the non-Hermiticity of the effective Hamiltonian in the context of this work might result in noncausality. However, similar to the CC Green’s function,^182^ we expect such issues to be of minor concern for single-reference states. The sum-over-state representability therefore represents a notable advantage compared to vertex corrections derived from approximate solutions of Hedin’s equations.^149,154,156,159,160,162,181,183–188^

In this work, we explore corrections to ***f*** through static Fock matrix corrections **Σ**(∞) as

55 $$\Sigma_{pq}\left(\infty \right)=\underset{rs}{\sum }\left\langle pq\right|\left|rs\right\rangle \left(\gamma_{pr}-\gamma_{pr}^{\mathrm{HF}}\right)$$

where ⟨*pq*||*rs*⟩ = ⟨*pq*|*rs*⟩ – ⟨*pq*|*sr*⟩, **γ** and **γ** ^HF^ denote the correlated and HF one-body density matrices. Therefore, the augmented Fock matrix, used in the EOM treatment or, equivalently, in the *GW* supermatrix, reads

56 $$f=f^{\mathrm{HF}}+\Sigma \left(\infty \right)$$

where ***f*** ^HF^ is the HF (or alternative mean-field) Fock matrix. Depending on the choice of **γ**, this modification allows for mimicking self-consistency effects^189–191^ (see Section 9), and/or additional vertex corrections beyond *GW*. Note that such static self-energy corrections to the density matrix naturally arise in alternative perturbative construction schemes of the self-energy.^86^

Beyond these static corrections, the ECC framework also enables the inclusion of missing exchange contributions in the matrices ***Ñ***, ***N***, and ***D***, which enter ***H*** ^EOM^ [see eq 60]. Here, we consider exchange-like contributions that arise from particle-hole contractions of the electron repulsion integrals ⟨*pq*|*rs*⟩ with the ECC amplitudes (***t*** and ***z***). The inclusion of such contributions shares similarity with the SOSEX ground-state energy correction to drCCD proposed in ref 192.

To illustrate how different exchange contributions can be included, we start investigating the diagrammatic representation of the following ring contraction between the electron repulsion integrals ⟨*pq*|*rs*⟩ with the ECC amplitude ***t***

57

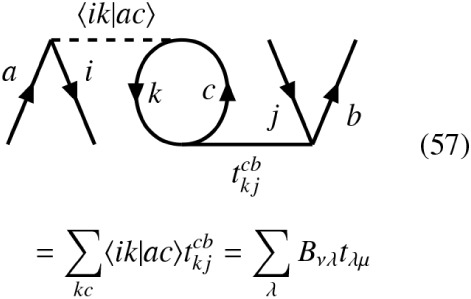

entering the ***D*** block of ***H*** ^EOM^ [see eq 60 and eq 65 and 66]. In this case, the particle and hole lines are connected to the same vertices of ⟨*ik*|*ac*⟩ and $t_{kj}^{cb}$ . Three distinct exchange contributions can be identified by exchanging the vertices to which the particle and hole lines are connected:

i Through connection to different vertices of the electron repulsion integral
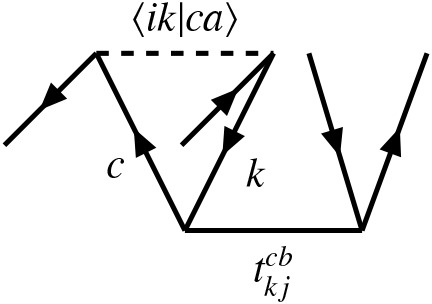

ii Through connection to different vertices of the amplitude ***t*** 
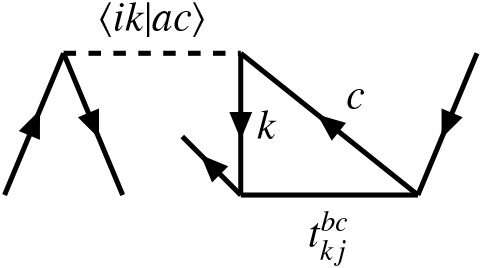

iii both
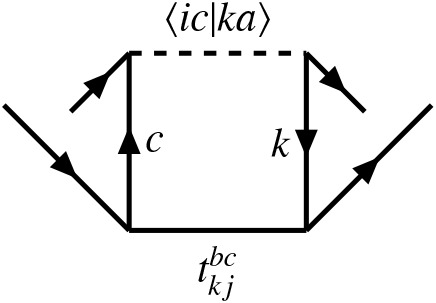

The latter can be identified as electron–hole-ladder/crossed-ring corrections.^113,193^ Additionally, we consider exchange contributions in (i) originating from the contraction with the excitation vector *R̂* (Section 6), i.e.,

58 $$\begin{array}{cc} & \underset{\nu }{\sum }A_{\nu ,\mu }r_{\mu j}=\underset{kc}{\sum }A_{ia,kc}r_{jkc}^{\left(m\right)}\\ & \to \underset{kc}{\sum }\left(A_{ia,kc}-\langle ic\left|ka\right\rangle \right)r_{jkc}^{\left(m\right)} \end{array}$$

The first term corresponds to the ring contraction
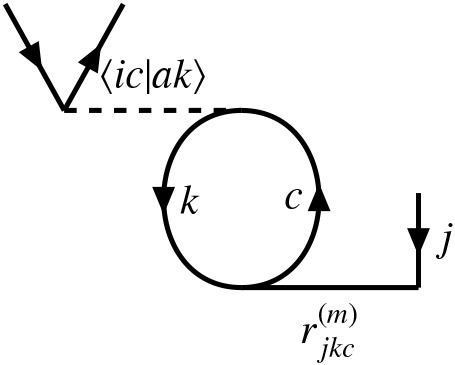


and the additional term in the last line represents the contraction of ⟨*ic*|*ka*⟩ with *r*_*jkc*_, where the particle-hole indices (*c* and *k*) are located at different vertices of the electron repulsion integral:
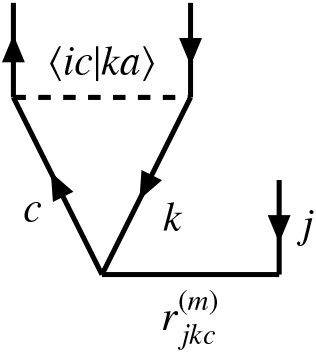
This example contributes to ***D*** [see eq 65].

To explore the importance of the different exchange corrections, we have implemented the underlying working equations for (i), (ii), and (iii) in spin-adapted form for the EOM effective Hamiltonian of eq 60. The resulting equations can be found in the Supporting Information. In the following, we will denote the different exchange corrections as $\Gamma_{V}^{x}$ for case (i), $\Gamma_{A}^{x}$ for case (ii), and $\Gamma_{\mathrm{CR}}^{x}$ for case (iii). Combinations of these corrections will be indicated by the addition of the respective labels, e.g., $\Gamma_{V+A}^{x}$ indicates the inclusion of both (i) and (ii) contributions.

We would like to stress that the proposed corrections (i) to (iii) are a choice made in the present work. The corrections are motivated also by their potential for low-scaling implementations. While the computational cost of the present approach is $O{(N}^{6})$ , we would like to emphasize that the cost for determining the amplitudes can be reduced to $O{(N}^{4})$ through techniques such as Cholesky decomposition.^112,165^ Furthermore, in combination with the resolution-of-the-identity (RI) technique,^194,195^ the matrix-vector products for the EOM eigenvalue problem, including exchange corrections, can be reduced to $O{(N}^{5})$ , whereas application of tensor-hypercontraction techniques (THC)^196,197^ reduces this scaling further to $O{(N}^{4})$ . The applicability of these low-scaling techniques within the vertex corrections presented here will be explored in future work.

Beyond that, the choice made here can also be rationalized by comparison to alternative *GW* vertex corrections proposed in the literature (see refs 149,154,156,159,160,162,181,183–188 ). Within Hedin’s equations [see eqs 1–5], vertex correction enter both the self-energy Σ [see eq 4] and the polarizability *P* [see eq 2]. Note that the vertex itself is defined through the functional derivative of Σ with respect to *G* [see eq 1]. This formal structure has motivated a variety of practical schemes, commonly classified according to whether vertex corrections are included in Σ, in *P*, or in both. Moreover, different approximations to the vertex Γ have been explored,^154,159,162,186,187^ many of which suffer from a lack of causality.^160,181,198^

The choices made in the present work can be interpreted as modifying both Σ and *P* such that the resulting (non-Hermitian) self-energy allows for a sum-over-state representation. However, several alternative, potentially systematically improvable, vertex corrections can be devised within the ECC framework, which we leave for future work. For example, of particular interest are the inclusion of additional contributions in the amplitude equations themselves and the effect of particle–particle and hole–hole ladder contributions at the EOM level.

## 9 *GW* Linearized Density Matrix from ECC Perturbation Theory

As noted in Section 8, the inclusion of static Fock matrix corrections **Σ**(∞) allows for mimicking self-consistency effects within the *GW* approximation. Here, we derive the *GW* linearized one-body density matrix **γ** within the ECC framework, which can be used to compute **Σ**(∞).

Refs 189–191 derived the linearized one-body density matrix within the *GW* approximation as

59a $$\gamma_{ij}=\delta_{ij}-\underset{a\nu }{\sum }\frac{M_{ia,\nu }M_{ja,\nu }}{\left(\epsilon_{i}-\epsilon_{a}-\Omega_{\nu }\right)\left(\epsilon_{j}-\epsilon_{a}-\Omega_{\nu }\right)}$$

59b $$\gamma_{ab}=\underset{i\nu }{\sum }\frac{M_{ai,\nu }M_{bi,\nu }}{\left(\epsilon_{i}-\epsilon_{a}-\Omega_{\nu }\right)\left(\epsilon_{i}-\epsilon_{b}-\Omega_{\nu }\right)}$$

59c $$\gamma_{ia}=\frac{f_{ia}}{\epsilon_{i}-\epsilon_{a}}+\frac{1}{\epsilon_{i}-\epsilon_{a}}\left[\underset{b\nu }{\sum }\frac{M_{ib,\nu }M_{ab,\nu }}{\epsilon_{i}-\epsilon_{b}-\Omega_{\nu }}+\underset{j\nu }{\sum }\frac{M_{ij,\nu }M_{aj,\nu }}{\epsilon_{j}-\epsilon_{a}-\Omega_{\nu }}\right]$$

In the following, we show how these equations can be derived within the ECC framework perturbatively. For this, we partition the Hamiltonian as

60 $$Ĥ\left(\lambda \right)={Ĥ}_{0}+\lambda \mathrm{V̂}$$

where λ denotes the perturbation parameter, and

61a $${Ĥ}_{0}=\underset{p}{\sum }f_{pp}{â}_{p}^{†}{â}_{p}+\underset{\mu \nu }{\sum }{\mathrm{A̿}}_{\mu \nu }{\mathrm{b̂}}_{\nu }^{†}{\mathrm{b̂}}_{\mu }$$

61b $$\mathrm{V̂}=\underset{pq\nu }{\sum }{\mathrm{M̿}}_{pq\nu }{â}_{p}^{†}{â}_{q}\left({\mathrm{b̂}}_{\nu }^{†}+{\mathrm{b̂}}_{\nu }\right)+\underset{p\neq q}{\sum }f_{pq}{â}_{p}^{†}{â}_{q}$$

Within perturbation theory,^199^ the doubly similarity-transformed Hamiltonian reads

62 $$\mathrm{H̿}\left(\lambda \right)=e^{Ẑ(\lambda )}e^{-\mathrm{T̂}(\lambda )}Ĥ\left(\lambda \right)e^{\mathrm{T̂}(\lambda )}e^{-Ẑ(\lambda )}$$

where the excitation and deexcitation operators read

63a $$\mathrm{T̂}\left(\lambda \right)=\lambda {\mathrm{T̂}}^{\left(1\right)}+\lambda^{2}{\mathrm{T̂}}^{\left(2\right)}+\cdot \cdot \cdot$$

63b $$Ẑ\left(\lambda \right)=\lambda {Ẑ}^{\left(1\right)}+\lambda^{2}{Ẑ}^{\left(2\right)}+\cdot \cdot \cdot$$

with

64a $${\mathrm{T̂}}^{\left(n\right)}={\mathrm{T̂}}_{1}^{\left(n\right)}+{\mathrm{T̂}}_{2}^{\left(n\right)}=\underset{ia}{\sum }t_{ia}^{\left(n\right)}{â}_{a}^{†}{â}_{i}+\underset{ia\nu }{\sum }t_{ia\nu }^{\left(n\right)}{â}_{a}^{†}{â}_{i}{\mathrm{b̂}}_{\nu }^{†}$$

64b $${Ẑ}^{\left(n\right)}={Ẑ}_{1}^{\left(n\right)}+{Ẑ}_{2}^{\left(n\right)}=\underset{ia}{\sum }z_{ia}^{\left(n\right)}{â}_{i}^{†}{â}_{a}+\underset{ia\nu }{\sum }z_{ia\nu }^{\left(n\right)}{\mathrm{b̂}}_{\nu }{â}_{i}^{†}{â}_{a}$$

and the *n*th-order amplitude equations can be deduced from

65a $${\mathrm{T̂}}_{k}^{\left(n\right)}\to \frac{1}{n!}\frac{\partial^{n}\left\langle k\right|\mathrm{H̿}\left(\lambda \right)\left|0\right\rangle }{\partial \lambda^{n}}{|}_{\lambda =0}=0$$

65b $${Ẑ}_{k}^{\left(n\right)}\to \frac{1}{n!}\frac{\partial^{n}\left\langle 0\right|\mathrm{H̿}\left(\lambda \right)\left|k\right\rangle }{\partial \lambda^{n}}{|}_{\lambda =0}=0$$

where *k* ∈ {1, 2}, $\left|1\right\rangle ={â}_{a}^{†}{â}_{i}\left|0_{e}0_{B}\right\rangle$ , $\left|2\right\rangle ={â}_{a}^{†}{â}_{i}{\mathrm{b̂}}_{\nu }^{†}\left|0_{e}0_{B}\right\rangle$ , ⟨1| = (|1⟩)^†^, ⟨2| = (|2⟩)^†^, and |0⟩ ≡ |0_e_0_B_⟩ denotes the reference ground-state wave function.

Within the perturbative expansion, the *n*th-order contribution to the one-body density matrix is obtained from

66 $$\gamma_{pq}^{\left(n\right)}=\frac{\partial E^{\left(n\right)}}{\partial f_{pq}}$$

Under certain approximations, the first-order density matrix contributions of the occupied-occupied and virtual–virtual blocks is obtained from the second-order energy expression

67 $$E^{\left(2\right)}=\left\langle 0\right|\left[\mathrm{V̂},{\mathrm{T̂}}^{\left(1\right)}\right]+\left[{Ẑ}^{\left(1\right)},\mathrm{V̂}\right]+\left[{Ẑ}^{\left(1\right)},\left[{Ĥ}_{0},{\mathrm{T̂}}^{\left(1\right)}\right]\right]\left|0\right\rangle$$

The resulting density matrix blocks coincide with the linearized one-body *GW* density matrix [see eq 76 and eq 77] only when canonical Hartree–Fock orbitals are employed. If this is not the case, additional terms, due to nonzero amplitudes $t_{ia}^{\left(1\right)}$ and $z_{ia}^{\left(1\right)}$ have to be considered in these blocks as well. Furthermore, only the first term of the occupied-virtual block [see eq 78] is obtained at second-order, which is zero for canonical orbitals.

The additional terms of eq 78 are recovered from a modified third-order energy expression

68 $$E^{\left(3\right)}=\left\langle 0\right|\left[\mathrm{V̂},{\mathrm{T̂}}_{1}^{\left(2\right)}\right]+\left[{Ẑ}_{1}^{\left(2\right)},\mathrm{V̂}\right]\left|0\right\rangle$$

Note that the resulting density matrix is not symmetric, i.e., γ_*pq*_ ≠ γ_*qp*_, and is symmetrized throughout this work for convenience. The natural occupation numbers from the perturbative ECC treatment then coincide with those obtained from the linearized one-body *GW* density matrix, as reported in eqs 76–78. Complete spin-adapted working equations are provided in the Supporting Information.

## 10 Computational Details

All calculations were performed using a custom implementation built on the PySCF package.^200,201^ Unless otherwise stated, *G*_0_ *W*_0_ calculations were carried out without invoking the diagonal approximation, and all results employ Hartree–Fock as the mean-field starting point. The linearized *GW* density matrix was computed using the perturbative ECC framework described in Section 9. The test set consists of 23 small molecules with accurate theoretical best estimates (TBEs) for inner- and outer-valence ionization potentials (IPs), taken from ref 202. All calculations employ the aug-cc-pVQZ basis set.^203,204^ We employ a large basis set to reduce basis set effects, thereby facilitating a more consistent, like-for-like comparison between the different schemes. The self-consistent field procedure is converged until the total energy changes by less than 10^–9^ *E*_h_. ECC amplitudes are iterated until the update satisfies ||Δ ***t*** || < 10^–8^ and ||Δ ***z*** || < 10^–8^. The Davidson solver used for computing *G*_0_ *W*_0_ quasiparticle energies is converged to 10^–8^ *E*_h_.

## 11 Numerical Results

First, we investigate the performance of various *G*_0_ *W*_0_ vertex corrections, as proposed in Section 8, for the calculation of the principal IPs (i.e., the lowest-energy IP of each system). The with mean absolute errors (MAEs) and mean-signed errors (MSEs) for these 23 principal IPs with respect to the reference TBE values for the benchmark set of ref 202 are displayed in Table 1. The relative errors are provided in the Supporting Information, and the error distributions are visualized in the violin plots of Figure 1.

**Figure 1:**
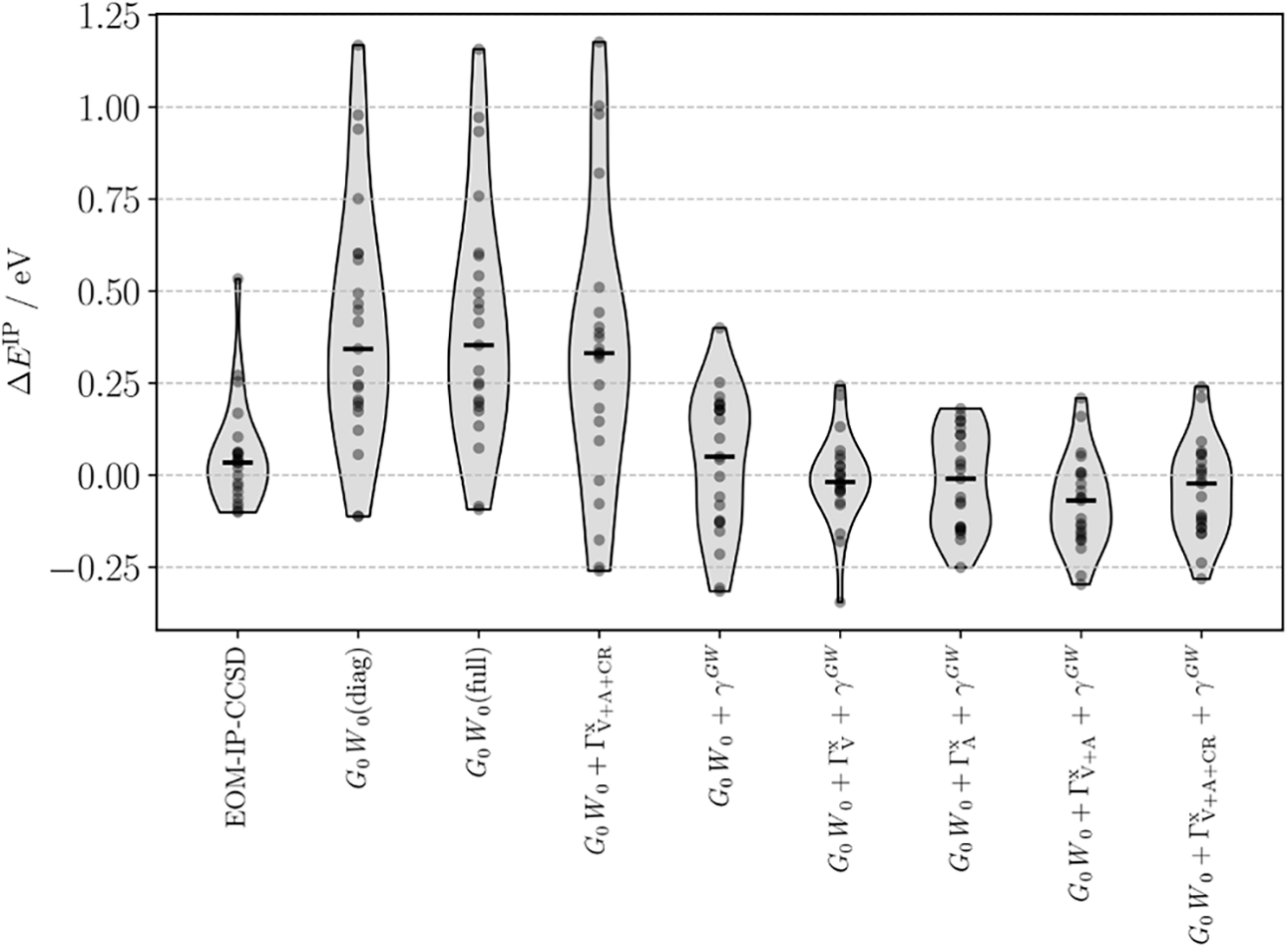
Violin plots of the errors (in eV) for the principal IPs obtained with various *G*_0_ *W*_0_ variants and EOM-IP-CCSD with respect to the TBEs for the benchmark set of 23 molecular systems from ref 202 computed with the aug-cc-pVQZ basis. Note that $\Gamma_{V+A}^{x}=\Gamma_{V}^{x}+\Gamma_{A}^{x}$ and $\Gamma_{V+A+\mathrm{CR}}^{x}=\Gamma_{V+A}^{x}+\Gamma_{\mathrm{CR}}^{x}$ .

**Table 1:** MAE and MSE (in eV) of the Principal IPs with Respect to the TBEs for the Benchmark Set of 23 Molecular Systems from Ref 202 ^a^

*G*_0_ *W*_0_ variants									
	CCSD^b^	(diag)^c^	(full)	$+\Gamma_{V+A+\mathrm{CR}}^{x}$	+ γ^*GW*^	$+\Gamma_{V}^{x}+\gamma^{GW}$	$\Gamma_{V+A}^{x}+\gamma^{GW}$	$+\Gamma_{V+A}^{x}+\gamma^{GW}$	$+\Gamma_{V+A+\mathrm{CR}}^{x}+\gamma^{GW}$
MAE	0.098	0.423	0.420	0.400	0.166	0.082	0.116	0.119	0.108
MSE	0.053	0.403	0.405	0.332	0.035	–0.011	–0.017	–0.076	–0.039

a Computed using various *G*_0_ *W*_0_ variants with the aug-cc-pVQZ basis.

b EOM-IP-CCSD data taken from ref 202.

c *G*_0_ *W*_0_ results computed within the diagonal approximation taken from ref 202.

In total, eight distinct *G*_0_ *W*_0_ variants are considered. First, *G*_0_ *W*_0_ IPs are computed within the diagonal approximation (as reported in ref 202, denoted as *G*_0_ *W*_0_ (diag), as well as using the full self-energy, denoted as *G*_0_ *W*_0_ (full). Furthermore, we consider *G*_0_ *W*_0_ calculations including static Fock matrix corrections using the *GW* linearized density matrix (denoted as +γ^*GW*^). Finally, we explore the effect of vertex corrections in ***H*** ^EOM^: (i) $\Gamma_{V}^{x}$ , (ii) $\Gamma_{A}^{x}$ , and (iii) $\Gamma_{\mathrm{CR}}^{x}$ . For a motivation of these contributions, the reader is referred to Section 8. Combinations of these vertex corrections are also considered and are denoted as, for example, $\Gamma_{V+A}^{x}$ , corresponding to $\Gamma_{V}^{x}+\Gamma_{A}^{x}$ . Vertex corrections are also considered in combination with static Fock matrix corrections.

Overall, similar errors in the principal IPs are observed for both *G*_0_ *W*_0_ (diag) and *G*_0_ *W*_0_ (full), MAEs of 0.423 and 0.420 eV, respectively. In general, the IPs for *G*_0_ *W*_0_ (diag) and *G*_0_ *W*_0_ (full) in combination with the Hartree–Fock starting point are systematically overestimated when compared to the TBEs. This is a known issue for *G*_0_ *W*_0_,^90,159,205–208^ and by performing full self-consistency, the IPs are known to be lowered.^90,159,205–207^ We observe a similar trend here, where the inclusion of static Fock matrix corrections based on the *GW* linearized density matrix lowers the IPs, resulting in a MAE of 0.166 eV. A similar trend has been observed in ref 209.

Including a combination of all three vertex corrections, $\Gamma_{V+A+\mathrm{CR}}^{x}$ , does only slightly improve the results, compared to *G*_0_ *W*_0_, and yields a MAE of 0.420 eV. However, when combined with static Fock matrix corrections, the MAE is significantly reduced. While all variants including vertex corrections in combination with static Fock matrix corrections $\left(+\Gamma_{V}^{x}+\gamma^{GW},+\Gamma_{A}^{x}+\gamma^{GW},+\Gamma_{V+A}^{x}+\gamma^{GW},+\Gamma_{V+A+\mathrm{CR}}^{x}+\gamma^{GW}\right)$ result in improvements when compared to *G*_0_ *W*_0_ + γ^*GW*^, the best performance is obtained when $\Gamma_{V}^{x}$ is considered. In this case one finds a MAE of 0.082 eV, and a MSE of −0.011 eV, which is even better than the MAE of 0.098 eV and MSE of −0.053 eV obtained from EOM-IP-CCSD. All variants lower the magnitude of the IPs slightly further when compared to *G*_0_ *W*_0_ + γ^*GW*^, while also reducing the spread of the errors. This reflects the limitations of HF orbitals as a starting point for the *G*_0_ *W*_0_ calculation. We conjecture that similar improvements could be achieved using optimally tuned range-separated hybrid orbitals.^210^

A direct comparison between the present results and previously proposed vertex corrections for molecular IPs is challenging. The available studies differ in several important aspects, including the molecular test sets considered,^159,181,186^ the underlying mean-field starting points,^184^ as well as the basis sets and other numerical settings employed.^154,186^ Even qualitative trends are difficult to establish: while some vertex-corrected schemes systematically decrease IPs,^162,186,208^ others lead to an overall increase.^159,184^ Recent evidence suggests that inclusion of vertex corrections only in *P* tends to reduce the quasiparticle gap and lower the IPs, whereas the inclusion of vertex corrections only in Σ tends to increase both the gap and the IPs.^160–162,185,188^ Achieving systematic improvements appears to require incorporating vertex corrections in both *P* and Σ.^161,162^ Consequently, it is not straightforward to provide a definitive assessment of the present vertex corrections relative to alternative approaches. Nevertheless, we emphasize that the vertex corrections introduced here lead to a clear and significant improvement in the description of principal IPs.

Next, we assess the performance of the different *G*_0_ *W*_0_ variants for the second IPs. The error distributions are visualized in the violin plots of Figure 2, and MAEs and MSEs are displayed in Table 2. The relative errors are provided in the Supporting Information. For the second IPs, we observe overall similar trends as for the principal IPs. While *G*_0_ *W*_0_ (diag) and *G*_0_ *W*_0_ (full) yield similar MAEs of 0.474 and 0.466 eV, the inclusion of static Fock matrix corrections through the *GW* linearized density reduces the MAE to 0.306 eV. Notably, all vertex corrections in combination with γ^*GW*^ yield significant improvements of more than 0.7 eV when compared to *G*_0_ *W*_0_ + γ^*GW*^. The best overall performance is achieved with $+\Gamma_{V+A+\mathrm{CR}}^{x}+\gamma^{GW}$ , resulting in a MAE of 0.183 eV, only slightly higher than the MAE of 0.157 eV obtained from EOM-IP-CCSD. Moreover, the corresponding MSE of 0.035 eV is lower than the MSE of 0.099 eV obtained for EOM-IP-CCSD. In all cases, the largest outlier is for Argon. Given the large reference TBE value for the second IP (29.182 eV), the relative error of 2.48% for EOM-IP-CCSD and 3.69% for $G_{0}W_{0}+\Gamma_{V+A+\mathrm{CR}}^{x}+\gamma^{GW}$ is relatively small compared to the absolute error of 0.725 and 1.078 eV, respectively.

**Figure 2:**
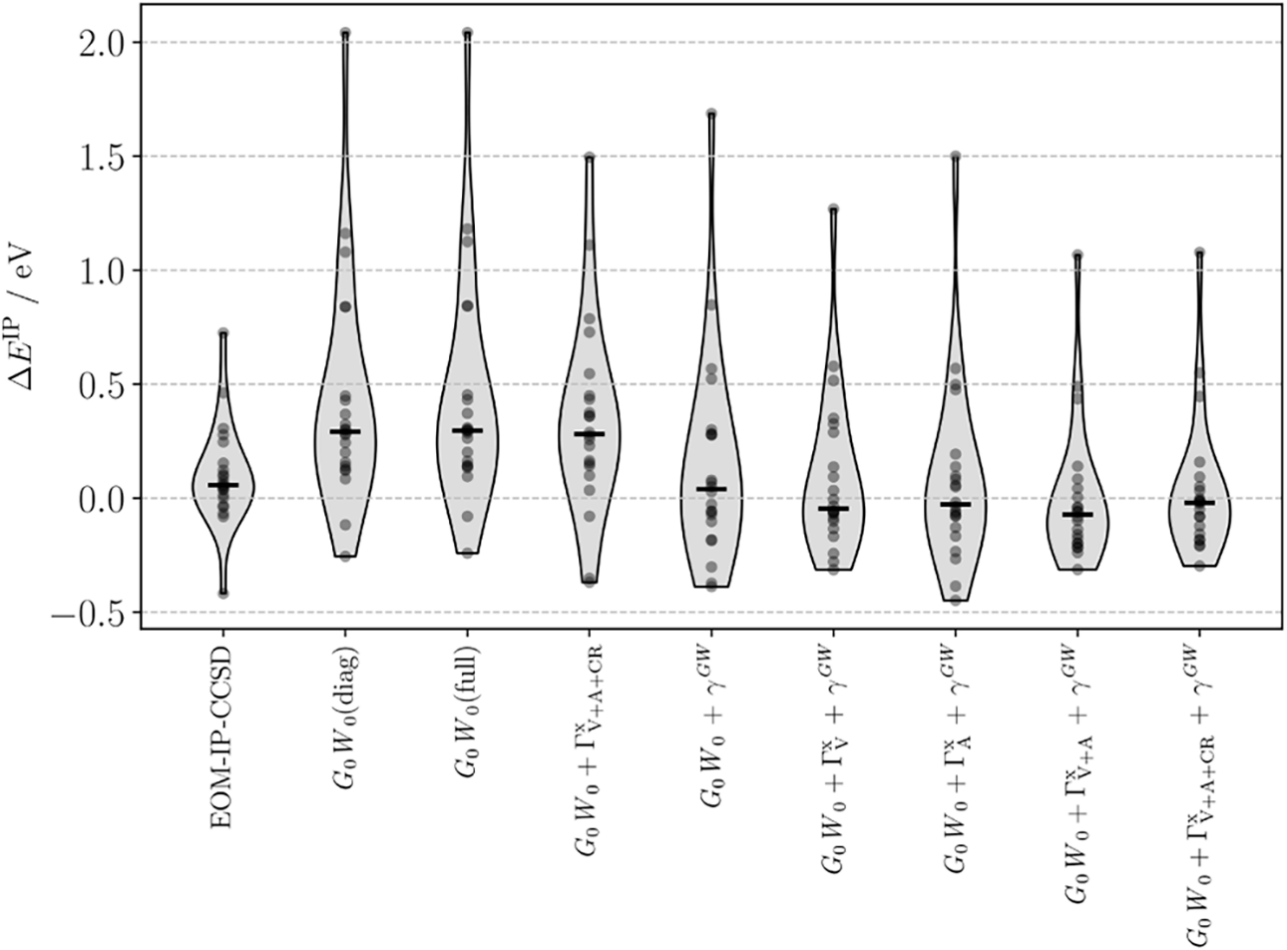
Violin plots of the errors (in eV) for the second IPs obtained with various *G*_0_ *W*_0_ variants and EOM-IP-CCSD with respect to the TBEs for the benchmark set of 23 molecular systems from ref 202 computed with the aug-cc-pVQZ basis. Note that $\Gamma_{V+A}^{x}=\Gamma_{V}^{x}+\Gamma_{A}^{x}$ and $\Gamma_{V+A+\mathrm{CR}}^{x}=\Gamma_{V+A}^{x}+\Gamma_{\mathrm{CR}}^{x}$ .

**Table 2:** MAE and MSE (in eV) of the Second IPs with Respect to the TBEs for the Benchmark Set of 23 Molecular Systems from Ref 202 ^a^

*G*_0_ *W*_0_ variants									
	CCSD^b^	(diag)^c^	(full)	$+\Gamma_{V+A+\mathrm{CR}}^{x}$	+ γ^*GW*^	$+\Gamma_{V}^{x}+\gamma^{GW}$	$\Gamma_{V+A}^{x}+\gamma^{GW}$	$+\Gamma_{V+A}^{x}+\gamma^{GW}$	$+\Gamma_{V+A+\mathrm{CR}}^{x}+\gamma^{GW}$
MAE	0.157	0.461	0.466	0.413	0.306	0.237	0.255	0.204	0.184
MSE	0.099	0.427	0.437	0.341	0.147	0.090	0.077	0.001	0.035

a Computed using various *G*_0_ *W*_0_ variants with the aug-cc-pVQZ basis.

b EOM-IP-CCSD data taken from ref 202.

c *G*_0_ *W*_0_ results computed within the diagonal approximation taken from ref 202.

## 12 Conclusion

In summary, we have established a formal connection between the extended direct ring coupled-cluster doubles (ECCD) framework and the *G*_0_ *W*_0_ approximation. When applied to the electron-boson Hamiltonian in combination with the equation-of-motion formalism, this procedure recovers the *G*_0_ *W*_0_ quasiparticle energies exactly. Furthermore, we have derived the linearized one-body *GW* density matrix within the ECC framework using perturbation theory. Exploiting the established connection between ECC and *GW*, we have proposed several vertex corrections beyond *GW* within the ECC framework, while retaining a sum-over-state presentation of the corresponding self-energy. Preliminary numerical results for a benchmark set of 23 small molecules demonstrate the potential of the proposed vertex corrections to significantly improve the accuracy of *G*_0_ *W*_0_ ionization potentials. Overall, the present work opens exciting avenues for including vertex corrections beyond *GW* within the ECC framework, such as the inclusion of particle–particle and hole–hole ladder contributions.

## Supplementary Material



## Notes

The derivations and working equations supporting the connection between the *GW* approximation and the extended coupled-cluster framework (key amplitude identities, the full spin-adapted EOM σ-vector equations, including the *G*_0_ *W*_0_–TDA subset and ECC-based vertex corrections, and the perturbative ECC formulation of the linearized one-body *GW* density matrix). It also reports the errors of the principal IPs with respect to the TBEs for the benchmark set of 23 molecular systems (PDF)

## References

[1] Crawford, T. D. ; Schaefer, H. F. An Introduction to Coupled Clus ter Theory for Computational Chemists. In Reviews in Computational Chemistry; Wiley, 2000; pp. 33–136.

[2] Piecuch P., Kowalski K., Pimienta I. S. O., Mcguire M. J. (2002). Recent advances
in electronic structure theory: Method of moments of coupled cluster
equations and renormalized coupled-cluster approaches. Int. Rev. Phys. Chem..

[3] Bartlett R. J., Musiał M. (2007). Coupled-clustertheoryin quantumchemistry. Rev. Mod. Phys..

[4] Shavitt, I. ; Bartlett, R. J. Many-Body Methods in Chemistry and Physics: mBPT and Coupled-Cluster Theory, Cambridge Molecular Science (Cam- bridge; University Press: Cambridge, 2009.

[5] Bartlett R. J. (2024). Perspective
on coupled-cluster theory. the evolution toward simplicity in quantum
chemistry. Phys. Chem. Chem. Phys..

[6] Rowe D. J. (1968). Equations-of-Motion
Method and the Extended Shell Model. Rev. Mod.
Phys..

[7] Koch H., Jensen H. J. A., Jorgensen P., Helgaker T. (1990). Excitation energies
from the coupled cluster singles and doubles linear response function
(CCSDLR). Applications to Be, CH+, CO, and H2O. J. Chem. Phys..

[8] Stanton J. F., Bartlett R. J. (1993). The equation of motion coupled-cluster method. a systematic
biorthogonal approach to molecular excitation ener- gies, transition
probabilities, and excited state properties. J. Chem. Phys..

[9] Koch H., Kobayashi R., Sanchez de Merás A., Jorgensen P. (1994). Calculation
of size-intensive transition moments from the coupled cluster singles
and doubles linear response function. J. Chem.
Phys..

[10] Sneskov K., Christiansen O. (2012). Excited state
coupled cluster methods. Wiley Interdiscip.
Rev.: comput. Mol. Sci..

[11] Tajti A., Szalay P. G., Császár A. G., Kállay M., Gauss J., Valeev E. F., Flowers B. A., Vázquez J., Stanton J. F. (2004). Heat: High accuracy extrapolated ab initio thermochemistry. J. Chem. Phys..

[12] Bomble Y. J., Vázquez J., Kállay M., Michauk C., Szalay P. G., Császár A. G., Gauss J., Stanton J. F. (2006). High-accuracy
extrapolated ab initio thermochemistry. II. Minor improvements to
the protocol and a vital simplification. J.
Chem. Phys..

[13] Harding M. E., Vazquez J., Ruscic B., Wilson A. K., Gauss J., Stanton J. F. (2008). High-accuracy extrapolated
ab initio thermochemistry.
iii. additional improvements and overview. J.
Chem. Phys..

[14] Karton A., Rabinovich E., Martin J. M. L., Ruscic B. (2006). W4 theory for computational
thermochemistry: In pursuit of confident sub-kj/mol predictions. J. Chem. Phys..

[15] Karton A., Sylvetsky N., Martin J. M. L. (2017). W4–17: A diverse and high-
confidence dataset of atomization energies for benchmarking high-level
electronic structure methods. J. Comput. Chem..

[16] Goerigk L., Grimme S. (2011). A thorough benchmark
of density functional methods
for general main group thermochemistry, kinetics, and noncovalent
interactions. Phys. Chem. Chem. Phys..

[17] Goerigk L., Grimme S. (2011). Efficient and accurate
double-hybrid-meta-gga density
functionals–evaluation with the extended gmtkn30 database for
general main group thermochemistry, kinetics, and noncovalent interac-
tions. J. Chem. Theory Comput..

[18] Goerigk L., Hansen A., Bauer C., Ehrlich S., Najibi A., Grimme S. (2017). A look at the density
functional theory zoo with the
advanced gmtkn55 database for general main group thermochemistry,
kinetics and noncovalent interactions. Phys.
Chem. Chem. Phys..

[19] Loos P.-F., Scemama A., Jacquemin D. (2020). The quest for highly accurate excitation
energies: A computational perspective. J. Phys.
Chem. Lett..

[20] Véril M., Scemama A., Caffarel M., Lipparini F., Boggio-Pasqua M., Jacquemin D., Loos P.-F. (2021). Questdb: A database
of highly accurate excitation energies for the electronic structure
community. Wiley Interdiscip. Rev.: comput.
Mol. Sci..

[21] Loos P.-F., Boggio-Pasqua M., Blondel A., Lipparini F., Jacquemin D. (2025). Quest database
of highly-accurate excitation energies. J. Chem.
Theory Comput..

[22] Hubbard J. (1957). The description
of collective motions in terms of many-body perturbation theory. Proc. R. Soc. London A.

[23] Coester F. (1958). Bound states
of a many-particle system. Nucl. Phys..

[24] Coester F., Kümmel H. (1960). Short-range
correlations in nuclear wave functions. Nucl.
Phys..

[25] Si̇nanoğlu O. (1962). Many-electron
theory of atoms and molecules. i. shells, electron pairs vs many-electron
correlations. J. Chem. Phys..

[26] Čížek J. (1966). On the Correlation
Problem in Atomic and Molecular Systems. Calculation of Wavefunction
Components in Ursell-Type Expansion Us- ing Quantum-Field Theoretical
Methods. J. Chem. Phys..

[27] Paldus J., Čížek J., Shavitt I. (1972). Correlation problems
in atomic and molecular systems. iv. extended coupled-pair many-electron
theory and its application to the bh 3 molecule. Phys. Rev. A.

[28] Bishop R. F. (1991). An overview
of coupled cluster theory and its applications in physics. Theor. Chem. Acc.

[29] Dean D. J., Hjorth-Jensen M. (2004). Coupled-cluster approach to nuclear physics. Phys. Rev. C.

[30] Kowalski K., Dean D. J., Hjorth-Jensen M., Papenbrock T., Piecuch P. (2004). Coupled cluster calculations of ground and excited
states of nuclei. Phys. Rev. Lett..

[31] Hagen G., Papenbrock T., Hjorth-Jensen M., Dean D. J. (2014). Coupled- cluster
computations of atomic nuclei. Rep. Prog. Phys..

[32] Gruber T., Liao K., Tsatsoulis T., Hummel F., Grüneis A. (2018). Applying the
coupled-cluster ansatz to solids and surfaces in the thermodynamic
limit. Phys. Rev..

[33] Zhang I. Y., Grüneis A. (2019). Coupled cluster theory in materials
science. Front. Mater..

[34] Wang X., Berkelbach T. C. (2020). Excitons
in solids from periodic equation- of-motion
coupled-cluster theory. J. Chem. Theory Comput..

[35] Neufeld V. A., Berkelbach T. C. (2023). Highly
accurate electronic structure of metallic solids
from coupled-cluster theory with nonperturbative triple excitations. Phys. Rev. Lett..

[36] Masios N., Irmler A., Schäfer T., Grüneis A. (2023). Averting the
infrared catastrophe in the gold standard of quantum chemistry. Phys. Rev. Lett..

[37] Ye H.-Z., Berkelbach T. C. (2024). Periodic
local coupled-cluster theory for insulators
and metals. J. Chem. Theory Comput..

[38] Vo E. A., Wang X., Berkelbach T. C. (2024). Performance
of periodic eom- ccsd
for bandgaps of inorganic semiconductors and insulators. J. Chem. Phys..

[39] Moerman E., Gallo A., Irmler A., Schäfer T., Hummel F., Grüneis A., Scheffler M. (2025). Finite-size
effects in periodic eom-ccsd for ionization energies and electron
affinities: Convergence rate and extrapolation to the thermodynamic
limit. J. Chem. Theory Comput..

[40] Moerman E., Miranda H., Gallo A., Irmler A., Schäfer T., Hummel F., Engel M., Kresse G., Scheffler M., Grüneis A. (2025). Exploring
the accuracy of the equation-of-motion coupled-cluster
band gap of solids. Phys. Rev. B.

[41] Purvis G. P., Bartlett R. J. (1982). A full coupled-cluster
singles and
doubles model: The inclusion of disconnected triples. J. Chem. Phys..

[42] Raghavachari K., Trucks G. W., Pople J. A., Head-Gordon M. (1989). A fifth-order
perturbation comparison of electron correlation theories. Chem. Phys. Lett..

[43] Bomble Y. J., Stanton J. F., Kállay M., Gauss J. (2005). Coupled-cluster methods
including noniterative corrections for quadruple excitations. J. Chem. Phys..

[44] Kodrycka M., Patkowski K. (2019). Platinum,
gold, and silver standards of intermolecular
interaction energy calculations. J. Chem. Phys..

[45] Helgaker T., Jørgensen P. (1988). Analytical calculation of geometrical deriva- tives
in molecular electronic structure theory. Adv.
Quantum Chem..

[46] Koch H., Jensen H. J. A., Jørgensen P., Helgaker T., Scuseria G. E., Schaefer H. F. (1990). Coupled cluster
energy derivatives. Analytic Hessian
for the closed-shell coupled cluster singles and doubles wave function:
Theory and applications. J. Chem. Phys..

[47] Koch H., Jørgensen P. (1990). Coupled cluster
response functions. J. Chem. Phys..

[48] Helgaker, T. ; Jørgensen, P. Calculation of geometrical derivatives in molecular electronic structure theory. In Methods in Computational Molec- ular Physics, Springer: Boston, MA, 1992, pp. 353–421.

[49] Gauss, J. Molecular properties.InModern Methods and Algorithms of Quantum Chemistry; Grotendorst, J. eds.; John von Neumann Institute for Computing, 2000pp. 541–592.

[50] Helgaker, T. ; Jørgensen, P. ; Olsen, J. Molecular Electronic-Structure Theory; Wiley, 2013.

[51] Hampe F., Stopkowicz S. (2019). Transition-dipole moments for electronic
excitations
in strong magnetic fields using equation-of-motion and linear response
coupled-cluster theory. J. Chem. Theory Comput..

[52] Musiał M., Kucharski S. A., Bartlett R. J. (2003). Equation-of-motion coupled cluster
method with full inclusion of the connected triple excitations for
ionized states: Ip-eom-ccsdt. J. Chem. Phys..

[53] Musiał M., Bartlett R. J. (2003). Equation-of-motion
coupled cluster method with full
inclusion of connected triple excitations for electron-attached states:
Ea-eom-ccsdt. J. Chem. Phys..

[54] Krylov A. I. (2008). Equation-of-motion
coupled-cluster methods for open-shell and electronically excited
species: The hitchhiker’s guide to fock space. Annu. Rev. Phys. Chem..

[55] Shen J., Piecuch P. (2013). Doubly electron-attached
and doubly ionized equation-of-motion
coupled-cluster methods with 4-particle–2-hole and 4-hole–2-particle
excitations and their active-space extensions. J. Chem. Phys..

[56] Gulania S., Jagau T.-C., Krylov A. I. (2019). Eom-cc
guide to fock-space travel:
the C2 edition. Faraday Discuss.

[57] Musial M., Bartlett R. J. (2008). Multireference Fock-space
coupled-cluster and equation-of-motion
coupled-cluster theories: The detailed interconnections. J. Chem. Phys..

[58] Musiał M., Perera A., Bartlett R. J. (2011). Multireference coupled-cluster theory:
The easy way. J. Chem. Phys..

[59] Lyakh D. I., Musiał M., Lotrich V. F., Bartlett R. J. (2012). Multireference nature
of chemistry: The coupled-cluster view. Chem.
Rev..

[60] Köhn A., Hanauer M., Mück L. A., Jagau T.-C., Gauss J. (2013). State- specific
multireference coupled-cluster theory. Wiley
Interdiscip. Rev.: comput. Mol. Sci..

[61] Krylov, A. I. The quantum chemistry of open-shell species. InReviews in Computational Chemistry. Parrill, A. L. ; Lipkowitz, K. B. pp. 151–224.Wiley, 2017

[62] Evangelista F. A. (2018). Perspective:
Multireference coupled cluster theories of dynamical electron correlation. J. Chem. Phys..

[63] Onida G., Reining L., Rubio A. (2002). Electronic
excitations: Density-functional
versus many-body green’s function approaches. Rev. Mod. Phys..

[64] Martin, R. M. ; Reining, L. ; Ceperley, D. M. Interacting Electrons: theory and Computational Approaches; Cambridge University Press, 2016.

[65] Salpeter E. E., Bethe H. A. (1951). A relativistic equation for bound-state problems. Phys. Rev..

[66] Hedin L. (1965). New method
for calculating the one-particle Green’s function with application
to the electron-gas problem. Phys. Rev..

[67] Aryasetiawan F., Gunnarsson O. (1998). The gw method. Rep. Prog. Phys..

[68] Reining L. (2017). The GW approximation:
Content, successes and limitations: The GW approximation. Wiley Interdiscip. Rev.: comput. Mol. Sci..

[69] Lundqvist B. I. (1967). Single-particle
spectrum of the degenerate electron gas: Ii. numerical results for
electrons coupled to plasmons. Physik der Kon-
Densierten Mater..

[70] Lundqvist B. I. (1967). Single-particle
spectrum of the degenerate electron gas: I. the structure of the spectral
weight function. Phys. Kondens. Mater..

[71] Mahan G. D., Sernelius B. E. (1989). Electron-electron
interactions and the bandwidth of
metals. Phys. Rev. Lett..

[72] Strinati G., Mattausch H. J., Hanke W. (1982). Dynamical aspects of
cor- relation
corrections in a covalent crystal. Phys. Rev.
B.

[73] Strinati G. (1982). Dynamical
shift and broadening of core excitons in semicon- ductors. Phys. Rev. Lett..

[74] Hybertsen M. S., Louie S. G. (1985). First-principles theory of quasiparticles: Calculation
of band gaps in semiconductors and insulators. Phys. Rev. Lett..

[75] Hybertsen M. S., Louie S. G. (1986). Electron correlation
in semiconductors and insulators:
Band gaps and quasiparticle energies. Phys.
Rev. B.

[76] Godby R. W., Schlüter M., Sham L. J. (1986). Accurate Exchange-Correlation Potential
for Silicon and Its Discontinuity on Addition of an Electron. Phys. Rev. Lett..

[77] Godby R. W., Schlüter M., Sham L. J. (1987). Quasiparticle energies
in GaAs and
AlAs. Phys. Rev. B.

[78] Schirmer, J. Many-Body Methods for Atoms, Molecules and Clusters; Springer, 2018.

[79] Dreuw A., Papapostolou A., Dempwolff A. L. (2023). Algebraic diagram- matic construction
schemes employing the intermediate state formalism: Theory, capabilities,
and interpretation. J. Phys. Chem. A.

[80] Cederbaum L. (1974). Application
of Green’s functions to excitations accompa- nying photoionization
in atoms and molecules. Mol. Phys..

[81] Schirmer J., Cederbaum L. S., Domcke W., von Niessen W. (1977). Strong Correlation
Effects in inner Valence Ionization of N2 AND CO. Chem. Phys..

[82] Cederbaum L. S., Schirmer J., Domcke W., von Niessen W. (1977). Complete breakdown
of the quasiparticle picture for inner valence electrons. J. Phys. B: at. Mol. Phys..

[83] von Niessen, W. ; Cederbaum, L. S. ; Domcke, W. On green’s function methods for the study of ionic states in atoms and molecules. InExcited States in Quantum Chemistry: theoretical and Experimental Aspects of the Electronic Structure and Properties of the Excited States in Atoms, Molecules and Solids; Nicolaides, C. A. ; Beck, D. R. ; Springer: Netherlands, Dordrecht, 1979pp. 183–272.

[84] Cederbaum L. S., Domcke W., Schirmer J., von Niessen W. (1980). Many- Body
Effects in Valence and Core Photoionization of Molecules. Phys. Scr.

[85] Von
Niessen W., Cederbaum L. S., Domcke W., Diercksen G. H. F. (1981). Green’s
function calculations on the complete valence ionization spectra of
HF, HCl, HBr AND HI. Chem. Phys..

[86] von
Niessen W., Schirmer J., Cederbaum L. (1984). Computational
methods for the one-particle green’s function. Comput. Phys. Rep..

[87] Cederbaum L. S. (1990). On green’s
functions and their applications. Int. J. Quantum
Chem..

[88] Shirley E. L., Martin R. M. (1993). Gw quasiparticle calculations in atoms. Phys. Rev. B.

[89] Rohlfing M., Louie S. G. (2000). Electron-hole excitations and optical
spectra from
first principles. Phys. Rev. B.

[90] Stan A., Dahlen N. E., van Leeuwen R. (2006). Fully self-consistent *gw* calculations for atoms and molecules. Europhys.
Lett..

[91] Rostgaard C., Jacobsen K. W., Thygesen K. S. (2010). Fully self-consistent *gw* calculations for molecules. Phys.
Rev. B.

[92] Blase X., Attaccalite C., Olevano V. (2011). First-principles GW calculations
for fullerenes, porphyrins, phtalocyanine, and other molecules of
interest for organic photovoltaic applications. Phys. Rev. B.

[93] Faber C., Attaccalite C., Olevano V., Runge E., Blase X. (2011). First- principles
GW calculations for DNA and RNA nucleobases. Phys. Rev. B.

[94] Ke S.-H. (2011). All-electron
gw methods implemented in molecular orbital space: Ionization energy
and electron affinity of conjugated molecules. Phys. Rev. B.

[95] Bruneval F. (2012). Ionization
energy of atoms obtained from gw self-energy or from random phase
approximation total energies. J. Chem. Phys..

[96] Bruneval F., Marques M. A. L. (2013). Benchmarking the Starting Points of the GW Approximation
for Molecules. J. Chem. Theory Comput..

[97] Golze D., Dvorak M., Rinke P. (2019). The gw compendium:
A practical guide
to theoretical photoemission spectroscopy. Front.
Chem..

[98] Marie, A. ; Ammar, A. ; Loos, P.-F. The GW approximation: A quan- tum chemistry perspective Advances In Quantum Chemistry, Novel Treatments Of Strong Correlations Elsevier 2024 90 157–184 10.1016/bs.aiq.2024.04.001

[99] Blase X., Duchemin I., Jacquemin D. (2018). The bethe–salpeter
equation
in chemistry: Relations with td-dft, applications and challenges. Chem. Soc. Rev..

[100] Blase X., Duchemin I., Jacquemin D., Loos P.-F. (2020). The bethe–salpeter
equation formalism: From
physics to chemistry. J. Phys. Chem. Lett..

[101] Watts J. D., Bartlett R. J. (1994). The inclusion of
connected triple
excitations in the equation-of-motion coupled-cluster method. J. Chem. Phys..

[102] Kamiya M., Hirata S. (2006). Higher-order equation-of-motion coupled-
cluster methods for ionization processes. J.
Chem. Phys..

[103] Gour J. R., Piecuch P. (2006). Efficient formulation and computer
implementation of the active-space electron-attached and ionized equation-of-motion
coupled-cluster methods. J. Chem. Phys..

[104] Lange M. F., Berkelbach T. C. (2018). On the Relation between Equation-of-
Motion Coupled-Cluster Theory and the GW Approximation. J. Chem. Theory Comput..

[105] Scuseria G. E., Scheiner A. C., Lee T. J., Rice J. E., Schaefer H. F. (1987). The closed-shell coupled cluster single and double
excitation (CCSD) model for the description of electron correlation.
A comparison with configuration interaction (CISD) results. J. Chem. Phys..

[106] Stanton J. F. (1993). Many-body
methods for excited state potential energy
surfaces. I. General theory of energy gradients for the equation-of-motion
coupled-cluster method. J. Chem. Phys..

[107] Noga J., Bartlett R. J. (1987). The full ccsdt model
for molecular
electronic structure. J. Chem. Phys..

[108] Scuseria G. E., Schaefer H. F. (1988). A new implementation
of the full
CCSDT model for molecular electronic structure. Chem. Phys. Lett..

[109] Kowalski K., Piecuch P. (2001). The active-space equation-of-motion
coupled- cluster methods for excited electronic states: Full EOMCCSDt. J. Chem. Phys..

[110] Kowalski K., Piecuch P. (2001). Excited-state potential energy curves
of CH+: A comparison of the EOMCCSDt and full EOMCCSDT results. Chem. Phys. Lett..

[111] Kucharski S. A., Włoch M., Musiał M., Bartlett R. J. (2001). Coupled-cluster theory for excited electronic states:
The full equation-of-motion coupled- cluster single, double, and triple
excitation method. J. Chem. Phys..

[112] Scuseria G. E., Henderson T. M., Sorensen D. C. (2008). The ground state
correlation energy of the random phase approximation from a ring coupled
cluster doubles approach. J. Chem. Phys..

[113] Scuseria G. E., Henderson T. M., Bulik I. W. (2013). Particle-particle
and quasiparticle random phase approximations: Connections to coupled
cluster theory. J. Chem. Phys..

[114] Freeman D. L. (1977). Coupled-cluster
expansion applied to the electron gas:
Inclusion of ring and exchange effects. Phys.
Rev. B.

[115] Jansen G., Liu R.-F., Ángyán J. G. (2010). On the
equivalence of ring-coupled cluster and adiabatic connection fluctuation-dissipation
theorem random phase approximation correlation energy expressions. J. Chem. Phys..

[116] Peng D., Steinmann S. N., van Aggelen H., Yang W. (2013). Equivalence of particle-particle
random phase approximation correlation
energy and ladder-coupled-cluster doubles. J.
Chem. Phys..

[117] Berkelbach T. C. (2018). Communication: Random-phase approximation excitation
energies from approximate equation-of-motion coupled-cluster doubles. J. Chem. Phys..

[118] Rishi V., Perera A., Bartlett R. J. (2020). A route
to improving
rpa excitation energies through its connection to equation-of-motion
coupled cluster theory. J. Chem. Phys..

[119] Quintero-Monsebaiz R., Monino E., Marie A., Loos P.-F. (2022). Connections
between many-body perturbation and coupled-cluster theories. J. Chem. Phys..

[120] Coveney C. J. N., Tew D. P. (2025). Diagrammatic theory
of the irreducible
coupled-cluster self-energy. Phys. Rev. B.

[121] Coveney C. J. N. (2025). Uncovering relationships between the electronic self-
energy and coupled-cluster doubles theory. J.
Phys. Chem. A.

[122] Coveney C. J. N., Tew D. P. (2026). Non-hermitian green’s
function
theory with n -body interactions: The coupled-cluster similarity transformation. Phys. Rev. Res..

[123] Bintrim S. J., Berkelbach T. C. (2021). Full-frequency
gw without frequency. J. Chem. Phys..

[124] Tölle J., Chan G. K.-L. (2023). Exact relationships between the gw
approximation and equation-of-motion coupled-cluster theories through
the quasi-boson formalism. J. Chem. Phys.

[125] Tölle J. (2025). Fully analytic
g0w0 nuclear gradients. J. Phys. Chem. Lett..

[126] Tölle J., Kitsaras M.-P., Loos P.-F. (2025). Fully analytic
nuclear
gradients for the bethe–salpeter equation. J. Phys. Chem. Lett..

[127] Kitsaras M.-P., Tölle J., Loos P.-F. (2026). Analytic g0w0 gradients
based on a double-similarity transformation equation-of-motion coupled-cluster
treatment. J. Chem. Phys..

[128] Arponen J. (1983). Variational principles and linked-cluster
exp s expansions
for static and dynamic many-body problems. Ann.
Phys..

[129] Arponen J. (1982). The method
of stationary cluster amplitudes and the
phase transition in the lipkin pseudospin model. J.Phys. G: nucl. Phys..

[130] Arponen J. S., Bishop R. F., Pajanne E. (1987). Extended coupled-cluster
method. i. generalized coherent bosonization as a mapping of quantum
theory into classical hamiltonian mechanics. Phys. Rev. A.

[131] Arponen J. S., Bishop R. F., Pajanne E. (1987). Extended coupled-cluster
method. ii. excited states and generalized random-phase approximation. Phys. Rev. A.

[132] Piecuch P., Bartlett R. J. (1999). Eomxcc: A new coupled-cluster method
for electronic excited states. Adv. Quantum
Chem..

[133] Fan P.-D., Kowalski K., Ppiecuch P. (2005). Non-iterative
corrections
to extended coupled-cluster energies employing the generalized method
of moments of coupled-cluster equations. Mol.
Phys..

[134] Fan P.-D., Piecuch P. (2006). The usefulness of exponential wave
function expansions employing one- and two-body cluster operators
in electronic structure theory: The extended and generalized coupled-cluster
methods. Adv. Quantum Chem..

[135] Pal S. (1986). Bivariational coupled-cluster approach for the study
of static elec-
tronic properties. Phys. Rev. A.

[136] Pal S. (1990). Coupled-cluster response approach:
Improved variational strategy. Phys. Rev. A.

[137] Ghose K. B., Nair P. G., Pal S. (1993). Implementation
of a
stationary coupled- cluster response method. Chem. Phys. Lett..

[138] Basu Kumar A., Vaval N., Pal S. (1998). An extended coupled-cluster
func- tional for molecular properties: Study of an analytical and
numerical ap- proach. Chem. Phys. Lett..

[139] Manohar P. U., Vaval N., Pal S. (2004). Extended coupled-cluster
approach for magnetizabilities of small molecules. Chem. Phys. Lett..

[140] Bartlett R. J., Noga J. (1988). The expectation value coupled-cluster
method and analytical energy derivatives. Chem.
Phys. Lett..

[141] Bartlett R. J., Kucharski S. A., Noga J. (1989). Alternative coupled-cluster
ansätze II. The unitary coupled-cluster method. Chem. Phys. Lett..

[142] Cooper B., Knowles P. J. (2010). Benchmark studies of variational,
unitary and extended coupled cluster methods. J. Chem. Phys..

[143] Evangelista F. A. (2011). Alternative single-reference coupled
cluster approaches
for multireference problems: The simpler, the better. J. Chem. Phys..

[144] Van Voorhis T., Head-Gordon M. (2000). The quadratic
coupled cluster doubles
model. Chem. Phys. Lett..

[145] Shirley E. L. (1996). Self-consistent gw and higher-order
calculations of
electron states in metals. Phys. Rev. B.

[146] Del Sole R., Reining L., Godby R. W. (1994). GwΓ
approximation
for electron self-energies in semiconductors and insulators. Phys. Rev. B.

[147] Schindlmayr A., Godby R. W. (1998). Systematic Vertex Corrections through
Iterative Solution of Hedin’s Equations Beyond the *GW* Approximation. Phys. Rev. Lett..

[148] Morris A. J., Stankovski M., Delaney K. T., Rinke P., García-González P., Godby R. W. (2007). Vertex corrections
in localized and extended systems. Phys. Rev.
B.

[149] Shishkin M., Marsman M., Kresse G. (2007). Accurate quasiparticle
spectra from self-consistent gw calculations with vertex corrections. Phys. Rev. Lett..

[150] Romaniello P., Guyot S., Reining L. (2009). The self-energy beyond
GW: Local and nonlocal vertex corrections. J.
Chem. Phys..

[151] Romaniello P., Bechstedt F., Reining L. (2012). Beyond the G W approximation:
Combining correlation channels. Phys. Rev. B.

[152] Grüneis A., Kresse G., Hinuma Y., Oba F. (2014). Ionization
potentials of solids: The importance of vertex corrections. Phys. Rev. Lett..

[153] Hung L., Bruneval F., Baishya K., Ogut S. (2017). Benchmarking
the GW Approximation and Bethe-Salpeter Equation for Groups IB and
IIB Atoms and Monoxides. J. Chem. Theory Comput..

[154] Maggio E., Kresse G. (2017). Gw vertex corrected
calculations
for molecular systems. J. Chem. Theory Comput..

[155] Wang Y., Rinke P., Ren X. (2021). Assessing
the *G*
_0_
*W*
_0_Γ_0_(1) Approach: Beyond *G*
_0_
*W*
_0_ with Hedin’s Full Second-Order Self-Energy
Contribution. J. Chem. Theory Comput..

[156] Mejuto-Zaera C., Vlček V. (2022). Self-consistency
in gw Γ formalism
leading to quasiparticle-quasiparticle couplings. Phys. Rev. B.

[157] Förster A., Visscher L. (2022). Exploring the statically
screened *G*
_3_
*W*
_2_ correction
to the GW self-energy: Charged excitations and total energies of finite
systems. Phys. Rev. B.

[158] Weng G., Mallarapu R., Vlček V. (2023). Embedding
vertex corrections in GW self-energy: Theory, implementation, and
outlook. Chem. Phys..

[159] Wen M., Abraham V., Harsha G., Shee A., Whaley K. B., Zgid D. (2024). Comparing self-consistent
GW and vertex-corrected *G*
_0_
*W*
_0_ (*G*
_0_
*W*
_0_ Γ) accuracy for molecular
ionization potentials. J. Chem. Theory Comput..

[160] Bruneval F., Förster A. (2024). Fully dynamic *G*
_3_
*W*
_2_ self-energy
for finite systems:
Formulas and benchmark. J. Chem. Theory Comput..

[161] Förster A., Bruneval F. (2024). Why does the GW approximation
give
accurate quasiparticle energies? The cancellation of vertex corrections
quantified. J. Phys. Chem. Lett..

[162] Förster A. (2025). Beyond quasi-particle self-consistent
GW for molecules
with vertex corrections. J. Chem. Theory Comput..

[163] Monino E., Loos P.-F. (2022). Unphysical discontinuities,
intruder
states and regularization in gw methods. J.
Chem. Phys..

[164] Monino E., Loos P.-F. (2023). Connections and performances of green’s
function methods for charged and neutral excitations. J. Chem. Phys..

[165] Tölle J., Kin-Lic Chan G. (2024). AB-g_0_ w_0_: A
practical g_0_ w_0_ method without frequency integration
based on an auxiliary boson expansion. J. Chem.
Phys..

[166] Scott C. J. C., Backhouse O. J., Booth G. H. (2023). A “moment-conserving”
reformulation of gw theory. J. Chem. Phys..

[167] Marie A., Loos P.-F. (2023). A Similarity Renormalization Group
Approach to Green’s Function Methods. J. Chem. Theory Comput..

[168] Lundqvist B. I. (1969). Characteristic structure in core electron spectra of
metals due to the electron-plasmon coupling. Phys. Kondens. Mater..

[169] Langreth D. C. (1970). Singularities
in the X-Ray Spectra of Metals. Phys. Rev. B.

[170] Hedin L. (1980). Effects of Recoil on Shake-Up Spectra
in Metals. Phys. Scr..

[171] Hedin L. (1999). On correlation effects in electron
spectroscopies and the gw approximation. J.
Phys.: condens. Matter..

[172] Ring, P. ; Schuck, P. The Nuclear Many-Body Problem; Springer, 2004.

[173] Henderson T. M., Bulik I. W., Stein T., Scuseria G. E. (2014). Seniority-based
coupled cluster theory. J. Chem. Phys..

[174] Henderson T. M., Bulik I. W., Scuseria G. E. (2015). Pair extended coupled
cluster doubles. J. Chem. Phys..

[175] Prasad M. D., Pal S., Mukherjee D. (1985). Some aspects
of self-consistent propagator theories. Phys.
Rev. A.

[176] Datta B., Mukhopadhyay D., Mukherjee D. (1993). Consistent
propagator theory based on the extended coupled-cluster parametrization
of the ground state. Phys. Rev. A.

[177] Mukherjee, D. ; Kutzelnigg, W. An effective liouvillean formalism for propagators in fock space: Connection with effective hamiltonian approach for energy differences. In Many-Body Methods in Quantum Chemistry: proceedings of the Symposium, Tel Aviv University 28–30 August 1988; Springer, 1989; pp. 257–274.

[178] Kim Y., Krylov A. I. (2023). Two algorithms for excited-state quantum solvers: Theory
and application to eom-uccsd. J. Phys. Chem.
A.

[179] Phillips J. T., Koulias L. N., Yuwono S. H., De Prince A. E. (2025). Comparing perturbative and commutator-rank-based truncation
schemes in unitary coupled-cluster theory. Mol.
Phys..

[180] Grazioli L., Kitsaras M.-P., Stopkowicz S. (2025). Unitary coupled-cluster
theory for the treatment of molecules in strong magnetic fields. J. Chem. Theory Comput..

[181] Bruneval F., Förster A., Pavlyukh Y. (2025). Gw+ 2sosex self-energy
made positive semidefinite. J. Chem. Theory
Comput..

[182] Nooijen M., Snijders J. G. (1992). Coupled cluster approach to the single-
particle green’s function. Int. J. Quantum
Chem..

[183] Chen W., Pasquarello A. (2015). Accurate band gaps of extended systems
via efficient vertex corrections in gw. Phys.
Rev. B.

[184] Ren X., Marom N., Caruso F., Scheffler M., Rinke P. (2015). Beyond the G W approximation: A second-order
screened exchange correction. Phys. Rev. B.

[185] Cunningham B., Grüning M., Azarhoosh P., Pashov D., Van Schilfgaarde M. (2018). Effect of
ladder diagrams on optical
absorption spectra in a quasi-particle self-consistent gw framework. Phys. Rev. Mater..

[186] Vlcek V. (2019). Stochastic
vertex corrections: Linear scaling methods for accurate
quasiparticle energies. J. Chem. Theory Comput..

[187] Rohlfing M. (2023). Approximate spatiotemporal structure
of the vertex
function *γ* (1, 2; 3) in many-body perturbation
theory. Phys. Rev. B.

[188] Cunningham B., Grüning M., Pashov D., Van Schilfgaarde M. (2023). Qs gw: Quasiparticle
self-consistent gw with ladder diagrams in w. Phys. Rev. B.

[189] Bruneval F. (2019). Improved density
matrices for accurate molecular ionization
potentials. Phys. Rev. B.

[190] Bruneval F. (2019). Assessment of the linearized gw density matrix for
molecules. J. Chem. Theory Comput..

[191] Bruneval F., Rodriguez-Mayorga M., Rinke P., Dvorak M. (2021). Improved one-shot
total energies from the linearized gw density matrix. J. Chem. Theory Comput..

[192] Grüneis A., Marsman M., Harl J., Schimka L., Kresse G. (2009). Making the random phase approximation
to electronic
correlation accurate. J. Chem. Phys..

[193] Orlando R., Romaniello P., Loos P.-F. (2023). The three channels
of many-body perturbation theory: GW , particle–particle, and
electron–hole T -matrix self-energies. J. Chem. Phys..

[194] Beebe N. H., Linderberg J. (1977). Simplifications
in the generation
and transformation of two-electron integrals in molecular calculations. Int. J. Quantum Chem..

[195] Pedersen T. B., Lehtola S., Galván I. F., Lindh R. (2024). The versatility of the cholesky decomposition in electronic structure
theory. Wiley Interdiscip. Rev.: comput. Mol.
Sci..

[196] Hohenstein E. G., Parrish R. M., Martínez T. J. (2012). Tensor
hypercontraction density fitting. I. Quartic scaling second- and third-order
Møller-Plesset perturbation theory. J.
Chem. Phys..

[197] Parrish R. M., Hohenstein E. G., Martínez T. J., Sherrill C. D. (2012). Tensor hypercontraction. II. least-squares
renormalization. J. Chem. Phys..

[198] Pavlyukh Y., Uimonen A.-M., Stefanucci G., van Leeuwen R. (2016). Vertex corrections
for positive-definite spectral functions
of simple metals. Phys. Rev. Lett..

[199] Kutzelnigg W. (2009). How many-body perturbation theory
(mbpt) has changed
quantum chemistry. Int. J. Quantum Chem..

[200] Sun Q., Berkelbach T. C., Blunt N. S., Booth G. H., Guo S., Li Z., Liu J., McClain J. D., Sayfutyarova E. R., Sharma S. (2018). Pyscf:
The python-based simulations of chemistry framework. Wiley Interdiscip. Rev.: comput. Mol. Sci..

[201] Sun Q., Zhang X., Banerjee S., Bao P., Barbry M., Blunt N. S., Bogdanov N. A., Booth G. H., Chen J., Cui Z.-H. (2020). Recent developments in the PySCF program package. J. Chem. Phys..

[202] Marie A., Loos P.-F. (2024). Reference Energies
for Valence Ionizations
and Satellite Transitions. J. Chem. Theory Comput..

[203] Dunning T. H. (1989). Gaussian basis
sets for use in correlated
molecular calculations. i. the atoms boron through neon and hydrogen. J. Chem. Phys..

[204] Woon D. E., Dunning T. H. (1993). Gaussian basis
sets for use in correlated molecular calculations. III. The atoms
aluminum through argon. J. Chem. Phys..

[205] Caruso F., Rinke P., Ren X., Scheffler M., Rubio A. (2012). Unified description of ground and
excited states of finite systems:
The self-consistent G W approach. Phys. Rev.
B.

[206] Caruso F., Rinke P., Ren X., Rubio A., Scheffler M. (2013). Self-consistent G W: All-electron
implementation with
localized basis functions. Phys. Rev. B.

[207] Caruso F., Dauth M., van Setten M. J., Rinke P. (2016). Benchmark of gw approaches for the gw100 test set. J. Chem. Theory Comput..

[208] Maggio E., Kresse G. (2016). Correlation energy for the homogeneous
electron gas: Exact bethe-salpeter solution and an approximate evaluation. Phys. Rev. B.

[209] Bruneval F., Dattani N., van Setten M. J. (2021). The gw
miracle in many-body perturbation theory for the ionization potential
of molecules. Front. Chem..

[210] McKeon C. A., Hamed S. M., Bruneval F., Neaton J. B. (2022). An optimally
tuned range-separated hybrid starting point for ab initio GW plus
Bethe–Salpeter equation calculations of molecules. J. Chem. Phys..

