# Supplementary material for: Connection between GW and Extended Coupled Cluster
Source: J Chem Theory Comput. 2026 Jun 29;22(13):6471–86. doi: 10.1021/acs.jctc.6c00378 (PMC13374021; doi:10.1021/acs.jctc.6c00378)
Supplement: Supplementary file 1 [file ct6c00378_si_001.pdf]

Supporting Information for "Connection between GW and Extended Coupled Cluster"

Johannes Tölle*,[1, a)] Marios-Petros Kitsaras,[2] Andreas Irmler,[3] Andreas Grüneis,[3] and Pierre-François Loos[2]

[1)] *Department of Chemistry, University of Hamburg, 22761 Hamburg, Germany; The Hamburg Centre for Ultrafast Imaging (CUI), Hamburg 22761, Germany*

[2)] *Laboratoire de Chimie et Physique Quantiques (UMR 5626), Université de Toulouse, CNRS, Toulouse, France*

[3)] *Institute for Theoretical Physics, TU Wien, Wiedner Hauptstraße 8-10/136, Vienna, Austria*

This Supporting Information provides the derivations and working equations supporting the connection between the GW approximation and the extended coupled-cluster (ECC) framework introduced in the main text. We present key amplitude identities, the full spin-adapted EOM σ-vector equations (including the G_0W_0–TDA subset and ECC-based vertex corrections), and the perturbative ECC formulation of the linearized one-body GW density matrix. It also reports the errors of the principal IPs with respect to the TBEs for the benchmark set of 23 molecular systems from Ref. 1 computed using various G_0W_0 variants with the aug-cc-pVQZ basis.

I. AMPLITUDE RELATIONS

To demonstrate the following relation

$$\mathbf{1} + \boldsymbol{z} + \boldsymbol{t}\cdot\boldsymbol{z} = (\mathbf{1} - \boldsymbol{t})^{-1} \tag{1}$$

we start by rewriting the left-hand side as

$$\mathbf{1} + \boldsymbol{z} + \boldsymbol{t}\cdot\boldsymbol{z} = \mathbf{1} + \boldsymbol{t}\cdot(\mathbf{1} - \boldsymbol{t}\cdot\boldsymbol{t})^{-1} + \boldsymbol{t}\cdot\boldsymbol{t}\cdot(\mathbf{1} - \boldsymbol{t}\cdot\boldsymbol{t})^{-1} \tag{2}$$

where we made use of[2]

$$\begin{aligned} \boldsymbol{z} &= \boldsymbol{Y}\cdot(\boldsymbol{X} - \boldsymbol{t}\cdot\boldsymbol{Y})^{-1} \\ &= \boldsymbol{t}\cdot(\mathbf{1} - \boldsymbol{t}\cdot\boldsymbol{t})^{-1} \end{aligned} \tag{3}$$

Realizing that the right-hand side of Eq. (2) can be recast into a von-Neumann series

$$\begin{aligned} &\mathbf{1} + \boldsymbol{t}\cdot(\mathbf{1} - \boldsymbol{t}\cdot\boldsymbol{t})^{-1} + \boldsymbol{t}\cdot\boldsymbol{t}\cdot(\mathbf{1} - \boldsymbol{t}\cdot\boldsymbol{t})^{-1} \\ &= \mathbf{1} + \sum_{k=0}^{\infty}\boldsymbol{t}^{2k+1} + \sum_{k=0}^{\infty}\boldsymbol{t}^{2k+2} \\ &= \sum_{k=0}^{\infty}\boldsymbol{t}^{k} = (\mathbf{1} - \boldsymbol{t})^{-1} \end{aligned} \tag{4}$$

establishes the relationship in Eq. (1).

II. σ-VECTOR EQUATIONS

The spin-adapted σ-vector equations for the mth excitation vector in the EOM eigenvalue problem defined in Eq. 46 for the doubly similarity-transformed electron-boson Hamiltonian, are presented in Sec. II A. The additional vertex corrections to the G_0W_0 approximation within the EOM-ECC framework are presented in Sec. II B.

The Einstein summation convention is implied over all repeated indices throughout. Electron repulsion integrals are defined in chemists' notation as $V_{pqrs} = (pq|rs)$. Furthermore, composite electron-hole fermionic indices, denoted as $\nu, \mu, \ldots$ in the manuscript, are resolved into occupied $(i, j, k, l, m, n, \ldots)$ and virtual $(a, b, c, d, e, f, \ldots)$ fermionic indices. Consquently, the amplitude tensors $\boldsymbol{t}$ and $\boldsymbol{z}$ are defined as $t_{\nu\mu} = t_{ijab}$ and $z_{\nu\mu} = z_{ijab}$, the excitation vectors as $r^{(m)}_{i\nu} = r^{(m)}_{ijb}$ and $r^{(m)}_{\nu a} = r^{(m)}_{jba}$.

A. EOM-ECC working equations

A subset of the full EOM-ECC σ-vector equations corresponds to the G_0W_0 σ-vector equations within the Tamm-Dancoff approximation (TDA). The equations were first presented in Ref. 3, and are provided here for completeness. The σ-vector equations for G_0W_0 within the TDA read

$$\sigma^{(m)}_{i} = f_{ij}r^{(m)}_{j} + f_{ia}r^{(m)}_{a} + 2V_{iabj}r^{(m)}_{jba} + 2V_{kijb}r^{(m)}_{kjb} \tag{5a}$$

$$\begin{aligned} \sigma^{(m)}_{ijb} &= V_{icbj}r^{(m)}_{c} + V_{jbki}r^{(m)}_{k} \\ &\quad - f_{bc}r^{(m)}_{ijc} + f_{ki}r^{(m)}_{kjb} + f_{lj}r^{(m)}_{ilb} - 2V_{lcbj}r^{(m)}_{ilc} \end{aligned} \tag{5b}$$

$$\sigma^{(m)}_{a} = f_{ab}r^{(m)}_{b} + f_{ai}r^{(m)}_{i} + 2V_{jbca}r^{(m)}_{jbc} + 2V_{iajb}r^{(m)}_{ijb} \tag{5c}$$

$$\begin{aligned} \sigma^{(m)}_{jba} &= V_{jbca}r^{(m)}_{c} + V_{jbia}r^{(m)}_{i} \\ &\quad + f_{ca}r^{(m)}_{jbc} + f_{cb}r^{(m)}_{jca} + f_{ij}r^{(m)}_{iba} + 2V_{jbck}r^{(m)}_{kca} \end{aligned} \tag{5d}$$

Next, the direct-ring ECC σ-vector equations containing the contraction with $\boldsymbol{t}$ and $\boldsymbol{z}$ are presented. The direct contractions with the amplitudes $\boldsymbol{t}$ read

$$\sigma^{(m)}_{i}[t] = 4V_{lcai}t_{lkcd}r^{(m)}_{kda} \tag{6a}$$

$$\begin{aligned} \sigma^{(m)}_{ijb}[t] &= 2V_{kcai}t_{kjcb}r^{(m)}_{a} + 2V_{laki}t_{ljab}r^{(m)}_{a} \\ &\quad - 4V_{lcka}t_{kjab}r^{(m)}_{ilc} \end{aligned} \tag{6b}$$

$$\sigma^{(m)}_{a}[t] = 4V_{icba}t_{ikcd}r^{(m)}_{kdb} \tag{6c}$$

$$\sigma^{(m)}_{jba}[t] = 4V_{jbic}t_{ikcd}r^{(m)}_{kda} \tag{6d}$$

while the direct contractions with the amplitudes $\boldsymbol{z}$ are

$$\sigma^{(m)}_{i}[z] = 4V_{raki}z_{lrca}r^{(m)}_{klc} \tag{7a}$$

$$\sigma^{(m)}_{a}[z] = 4V_{kabi}z_{licb}r^{(m)}_{klc} \tag{7b}$$

$$\sigma^{(m)}_{jba}[z] = 2V_{scda}z_{jsbc}r^{(m)}_{d} + 2V_{kacs}z_{jsbc}r^{(m)}_{k} \tag{7c}$$

a) Electronic mail: johannes.toelle@uni-hamburg.de

Lastly, the direct-ring contractions containing both $\boldsymbol{t}$ and $\boldsymbol{z}$ amplitudes are given by

$$\sigma_i^{(m)}[t,z] = 8V_{kcmi}z_{jled}t_{klcd}r_{mje}^{(m)} \tag{8a}$$

$$\sigma_a^{(m)}[t,z] = 8V_{makc}z_{jled}t_{klcd}r_{mje}^{(m)} \tag{8b}$$

$$\sigma_{jba}^{(m)}[t,z] = 4V_{kcea}z_{jlbd}t_{klcd}r_e^{(m)} + 4V_{iakc}z_{jlbd}t_{klcd}r_i^{(m)} \tag{8c}$$

B. Vertex corrections within EOM-ECC

As explained in the manuscript (Sec. VIII), the direct-ring σ-vector equations are modified to include exchange contributions in three distinct ways:

(i) Exchange corrections in the two-electron integrals ($\Gamma_{\mathrm{V}}^{\mathrm{x}}$):

$$\sigma_i^{(m)} = -V_{ibaj}r_{jba}^{(m)} - V_{kbji}r_{kjb}^{(m)} \tag{9a}$$

$$\sigma_{ijb}^{(m)} = V_{ljbc}r_{ilc}^{(m)} \tag{9b}$$

$$\sigma_a^{(m)} = -V_{jcba}r_{jbc}^{(m)} - V_{ibja}r_{ijb}^{(m)} \tag{9c}$$

$$\sigma_{jba}^{(m)} = -V_{jckb}r_{kca}^{(m)} \tag{9d}$$

$$\sigma_i^{(m)}[t] = -2V_{liac}t_{lkcd}r_{kda}^{(m)} \tag{9e}$$

$$\sigma_{ijb}^{(m)}[t] = -V_{kipc}t_{kjcb}r_p^{(m)} - V_{kali}t_{ljab}r_j^{(m)} + 2V_{lakc}t_{kjab}r_{ilc}^{(m)} \tag{9f}$$

$$\sigma_a^{(m)}[t] = -2V_{iabc}t_{ikcd}r_{kdb}^{(m)} \tag{9g}$$

$$\sigma_{jba}^{(m)}[t] = -2V_{jcib}t_{ikcd}r_{kda}^{(m)} \tag{9h}$$

$$\sigma_i^{(m)}[z] = -2V_{ibkj}z_{ljcb}r_{klc}^{(m)} \tag{9i}$$

$$\sigma_a^{(m)}[z] = -2V_{kjba}z_{ljcb}r_{klc}^{(m)} \tag{9j}$$

$$\sigma_{jba}^{(m)}[z] = -V_{icda}z_{jibd}r_c^{(m)} - V_{kida}z_{jibd}r_k^{(m)} \tag{9k}$$

$$\sigma_i^{(m)}[z,t] = -4V_{mcki}z_{jled}t_{klcd}r_{mje}^{(m)} \tag{9l}$$

$$\sigma_a^{(m)}[z,t] = -4V_{mcka}z_{jled}t_{klcd}r_{mje}^{(m)} \tag{9m}$$

$$\sigma_{jba}^{(m)}[z,t] = -2V_{kace}z_{jlbd}t_{klcd}r_e^{(m)} - 2V_{icka}z_{jlbd}t_{klcd}r_i^{(m)} \tag{9n}$$

(ii) Exchange corrections in the amplitudes ($\Gamma_{\mathrm{A}}^{\mathrm{x}}$):

$$\sigma_i^{(m)}[t] = -2V_{ldai}t_{lkcd}r_{kca}^{(m)} \tag{10a}$$

$$\sigma_{ijb}^{(m)}[t] = -V_{kdai}t_{kjbd}r_a^{(m)} - V_{lcki}t_{ljbc}r_k^{(m)} + 2V_{lckd}t_{kjbd}r_{ilc}^{(m)} \tag{10b}$$

$$\sigma_a^{(m)}[t] = -2V_{idba}t_{ikcd}r_{kcb}^{(m)} \tag{10c}$$

$$\sigma_{jba}^{(m)}[t] = -2V_{jbid}t_{ikcd}r_{kca}^{(m)} \tag{10d}$$

$$\sigma_i^{(m)}[z] = -2V_{jbki}z_{ljcb}r_{klc}^{(m)} \tag{10e}$$

$$\sigma_a^{(m)}[z] = -2V_{kabj}z_{ljbc}r_{klc}^{(m)} \tag{10f}$$

$$\sigma_{jba}^{(m)}[z] = -V_{lcda}z_{jlcb}r_d^{(m)} - V_{kacl}z_{jlcb}r_k^{(m)} \tag{10g}$$

(iii) Exchange corrections in both ($\Gamma_{\mathrm{CR}}^{\mathrm{x}}$):

$$\sigma_i^{(m)}[t] = V_{liad}t_{lkcd}r_{kca}^{(m)} \tag{11a}$$

$$\sigma_{ijb}^{(m)}[t] = V_{kjad}t_{kibd}r_a^{(m)} - V_{kclj}t_{libc}r_k^{(m)} - V_{ldkc}t_{kjbd}r_{ilc}^{(m)} \tag{11b}$$

$$\sigma_a^{(m)}[t] = V_{iabd}t_{ikcd}r_{kcb}^{(m)} \tag{11c}$$

$$\sigma_{jba}^{(m)}[t] = V_{jdib}t_{ikcd}r_{kca}^{(m)} \tag{11d}$$

$$\sigma_i^{(m)}[z] = -2V_{jbki}z_{ljcb}r_{klc}^{(m)} \tag{11e}$$

$$\sigma_a^{(m)}[z] = -2V_{kabj}z_{ljbc}r_{klc}^{(m)} \tag{11f}$$

$$\sigma_{jba}^{(m)}[z] = -V_{lcda}z_{jlcb}r_d^{(m)} - V_{kacl}z_{jlcb}r_k^{(m)} \tag{11g}$$

$$\sigma_i^{(m)}[t,z] = -4V_{mcki}z_{jled}t_{klcd}r_{mje}^{(m)} \tag{11h}$$

$$\sigma_a^{(m)}[t,z] = -4V_{mcka}z_{jled}t_{klcd}r_{mje}^{(m)} \tag{11i}$$

$$\sigma_{jba}^{(m)}[t,z] = -2V_{kace}z_{jlbd}t_{klcd}r_e^{(m)} - 2V_{icka}z_{jlbd}t_{klcd}r_i^{(m)} \tag{11j}$$

III. LINEARIZED DENSITY MATRIX

The amplitude equations (Sec. IX) for the linearized one-body GW density matrix within the ECC perturbation theory framework are

$$0 = V_{ia,\nu}\left(\delta_{\nu,\mu} + 2t_{\nu,\mu}\right) + A_{\mu,\nu}t_{ia,\nu}^{(1)} + f_{ab}t_{ib,\mu}^{(1)} - f_{ji}t_{ja,\mu}^{(1)} + 2B_{\mu,\nu}t_{\nu,\lambda}t_{ia,\lambda}^{(1)} \tag{12}$$

for $t_{ia,\mu}^{(1)}$, and

$$0 = f_{ia} + f_{ab}t_{ib}^{(2)} - f_{ji}t_{ja}^{(2)} - 2V_{ij,\nu}\left(\delta_{\nu,\mu} + 2z_{\nu,\mu} + 4t_{\nu,\lambda}z_{\lambda,\mu}\right)t_{ja,\mu}^{(1)} + 2V_{ab,\nu}\left(\delta_{\nu,\mu} + 2z_{\nu,\mu} + 4t_{\nu,\lambda}z_{\lambda,\mu}\right)t_{ib,\mu}^{(1)} \tag{13}$$

for $t_{ia}^{(2)}$. The elements $A_{\nu,\mu}$ are defined as

$$A_{\nu,\mu} = A_{ia,jb} = \delta_{ij}f_{ab} - \delta_{ab}f_{ij} + B_{ia,jb}, \tag{14}$$

with $B_{\mu,\nu} = B_{ia,jb} = 2V_{iajb}$. The amplitude equations for $z_{ia,\mu}^{(1)}$ read

$$0 = V_{ia,\nu}\left(\delta_{\nu,\mu} + 2z_{\nu,\mu} + 4t_{\nu,\lambda}z_{\lambda,\mu}\right) + A_{\mu,\nu}z_{ia,\nu}^{(1)} + f_{ab}z_{ib,\mu}^{(1)} - f_{ji}z_{ja,\mu}^{(1)} + 2B_{\mu,\nu}t_{\nu,\lambda}z_{ia,\lambda}^{(1)} \tag{15}$$

and $z_{ia}^{(2)}$ is obtained from

$$0 = f_{ia} + f_{ab}z_{ib}^{(2)} - f_{ji}z_{ja}^{(2)} - 2V_{ij,\nu}\left(\delta_{\nu,\mu} + 2t_{\nu,\mu}\right)z_{ja,\mu}^{(1)} + 2V_{ab,\nu}\left(\delta_{\nu,\mu} + 2t_{\nu,\mu}\right)z_{ib,\mu}^{(1)} \tag{16}$$

Based on the above equations, the linearized one-body GW density matrix within ECC is computed as $\boldsymbol{\gamma}^{GW}$

$$\boldsymbol{\gamma}^{GW} = \boldsymbol{\gamma}^{\dagger} + \boldsymbol{\gamma}, \tag{17}$$

with

$$\gamma_{ij} = 2\delta_{ij} - 2t^{(1)}_{ja,\mu} z^{(1)}_{ia,\mu} \tag{18a}$$

$$\gamma_{ab} = 2t^{(1)}_{ia,\mu} z^{(1)}_{ib,\mu} \tag{18b}$$

$$\gamma_{ia} = t^{(2)}_{ia} \tag{18c}$$

$$\gamma_{ai} = z^{(2)}_{ia} \tag{18d}$$

We have ensured that the resulting natural occupation numbers from $\boldsymbol{\gamma}^{GW}$ within the ECC framework are identical to those obtained from the linearized GW density matrix presented in Refs. 4–6 [see Eqs. (59a)-(59c)].

IV. RELATIVE ERROR OF INNER- AND OUTER-VALENCE IONIZATION POTENTIALS (IPS)

The error relative errors for the principal and second IPs with respect to the TBEs for the benchmark set of 23 molecular systems from Ref. 1 are displayed in Table S1 and Table S2, respectively.

REFERENCES

[1] A. Marie and P.-F. Loos, "Reference Energies for Valence Ionizations and Satellite Transitions," J. Chem. Theory Comput. **20**, 4751–4777 (2024).

[2] M.-P. Kitsaras, J. Tölle, and P.-F. Loos, "Analytic g0w0 gradients based on a double-similarity transformation equation-of-motion coupled-cluster treatment," J. Chem. Phys. **164**, 044122 (2026).

[3] S. J. Bintrim and T. C. Berkelbach, "Full-frequency gw without frequency," J. Chem. Phys. **154**, 041101 (2021).

[4] F. Bruneval, "Improved density matrices for accurate molecular ionization potentials," Phys. Rev. B **99**, 041118 (2019).

[5] F. Bruneval, "Assessment of the linearized gw density matrix for molecules," J. Chem. Theory Comput. **15**, 4069–4078 (2019).

[6] F. Bruneval, M. Rodriguez-Mayorga, P. Rinke, and M. Dvorak, "Improved one-shot total energies from the linearized gw density matrix," J. Chem. Theory Comput. **17**, 2126–2136 (2021).

TABLE S1. Errors (in eV) of the principal IPs with respect to the TBEs for the benchmark set of 23 molecular systems from Ref. 1 computed using various G_0W_0 variants with the aug-cc-pVQZ basis.

		G_0W_0 variants							
Mol.	CCSD[a]	(diag)[b]	(full)	+ Γ^{x}_{A+V+CR}	+ γ^{GW}	+Γ^{x}_{V} + γ^{GW}	Γ^{x}_{A} + γ^{GW}	+Γ^{x}_{V+A} + γ^{GW}	+Γ^{x}_{V+A+CR} + γ^{GW}
H_2S	0.034	0.204	0.204	0.375	-0.152	-0.040	-0.150	-0.042	0.017
CS	0.254	1.168	1.157	1.177	0.176	0.217	0.128	0.160	0.212
BeO	-0.077	-0.111	-0.085	-0.251	0.043	-0.019	-0.072	-0.151	-0.121
CH_4	0.021	0.465	0.468	0.318	-0.004	-0.159	-0.009	-0.176	-0.158
C_2	0.533	0.585	0.596	1.003	-0.315	-0.180	-0.159	0.007	0.091
BN	-0.001	-0.112	-0.093	-0.015	-0.306	-0.345	-0.25	-0.273	-0.238
Ne	-0.101	0.122	0.134	-0.176	0.196	0.054	0.037	-0.132	-0.115
HF	-0.097	0.239	0.251	-0.078	0.190	0.025	0.029	-0.167	-0.142
CO_2	0.056	0.602	0.603	0.442	0.152	0.024	0.079	-0.069	-0.016
LiF	-0.045	0.056	0.074	-0.260	0.191	0.044	0.017	-0.158	-0.143
HCl	0.042	0.197	0.197	0.332	-0.130	-0.045	-0.140	-0.062	0.002
CH_2O	-0.030	0.602	0.542	0.182	0.252	-0.003	0.146	-0.138	-0.109
Ar	0.063	0.187	0.187	0.326	-0.126	-0.036	-0.144	-0.061	0.010
SiH_4	0.059	0.494	0.496	0.386	0.178	0.067	0.181	0.060	0.064
BH_3	0.043	0.448	0.449	0.331	0.100	-0.025	0.109	-0.024	-0.023
H_2O	-0.062	0.343	0.353	0.146	0.050	-0.045	-0.078	-0.198	-0.158
NH_3	-0.022	0.417	0.414	0.344	-0.059	-0.074	-0.141	-0.176	-0.128
BF	0.104	0.245	0.245	0.510	-0.215	0.001	-0.175	0.052	0.058
LiCl	0.060	0.173	0.175	0.245	-0.123	-0.080	-0.150	-0.118	-0.058
CO	0.271	0.940	0.934	0.981	0.176	0.243	0.148	0.209	0.241
F_2	-0.086	0.751	0.758	0.094	0.400	0.007	0.162	-0.296	-0.281
PH_3	0.034	0.283	0.284	0.402	-0.082	-0.021	-0.060	-0.000	0.036
N_2	0.168	0.978	0.972	0.820	0.213	0.132	0.110	0.007	0.056

[a] EOM-IP-CCSD data taken from Ref. 1.

[b] G_0W_0 results computed within the diagonal approximation taken from Ref. 1.

TABLE S2. Errors (in eV) of the second IPs with respect to the TBEs for the benchmark set of 23 molecular systems from Ref. 1 computed using various G_0W_0 variants with the aug-cc-pVQZ basis.

		G_0W_0 variants							
Mol.	CCSD[a]	(diag)[b]	(full)	+ Γ^{x}_{A+V+CR}	+ γ^{GW}	+Γ^{x}_{V} + γ^{GW}	Γ^{x}_{A} + γ^{GW}	+Γ^{x}_{V+A} + γ^{GW}	+Γ^{x}_{V+A+CR} + γ^{GW}
H_2S	0.037	0.298	0.296	0.362	-0.072	-0.060	-0.065	-0.058	-0.011
CS	0.123	0.127	0.141	0.449	-0.301	-0.168	-0.234	-0.099	-0.016
BeO	-0.033	0.085	0.095	-0.369	0.284	0.033	0.082	-0.199	-0.181
CH_4	0.278	0.840	0.842	0.728	0.300	0.289	0.193	0.140	0.159
BN	0.013	-0.117	-0.080	0.258	-0.388	-0.134	-0.386	-0.160	-0.049
Ne	0.154	-0.255	-0.241	0.035	-0.181	0.350	-0.448	0.084	0.093
HF	-0.081	0.157	0.144	-0.080	0.069	-0.070	-0.019	-0.176	-0.157
CO_2	0.462	1.079	1.125	1.110	0.567	0.578	0.497	0.489	0.549
LiF	-0.039	0.121	0.134	-0.351	0.277	-0.006	0.097	-0.216	-0.210
HCl	0.054	0.303	0.297	0.358	-0.060	-0.057	-0.056	-0.060	-0.002
CH_2O	0.049	0.141	0.162	0.153	-0.186	-0.243	-0.166	-0.238	-0.205
Ar	0.725	2.042	2.042	1.496	1.687	1.268	1.500	1.067	1.078
SiH_4	0.247	0.839	0.845	0.787	0.523	0.516	0.476	0.436	0.446
BH_3	0.096	0.448	0.453	0.434	0.079	0.094	0.050	0.042	0.051
H_2O	-0.067	0.323	0.309	0.099	0.029	-0.088	-0.079	-0.217	-0.185
NH_3	0.000	0.368	0.372	0.142	-0.057	-0.279	-0.077	-0.313	-0.297
BF	-0.418	0.285	0.302	0.165	0.279	0.137	0.138	0.004	0.032
LiCl	0.061	0.201	0.202	0.231	-0.102	-0.101	-0.128	-0.137	-0.080
CO	0.075	0.244	0.263	0.289	-0.028	-0.062	-0.036	-0.084	-0.024
F_2	0.104	1.161	1.181	0.274	0.848	0.326	0.569	-0.043	-0.081
PH_3	0.041	0.430	0.432	0.375	0.050	-0.035	0.056	-0.038	-0.013
N_2	0.306	0.278	0.286	0.546	-0.373	-0.314	-0.266	-0.197	-0.122

[a] EOM-IP-CCSD data taken from Ref. 1.

[b] G_0W_0 results computed within the diagonal approximation taken from Ref. 1.
